# Antiviral activities of *Schizonepeta tenuifolia* Briq. against enterovirus 71 *in vitro* and *in vivo*

**DOI:** 10.1038/s41598-017-01110-x

**Published:** 2017-04-20

**Authors:** Sin-Guang Chen, Mei-Ling Cheng, Kuan-Hsing Chen, Jim-Tong Horng, Ching-Chuan Liu, Shih-Min Wang, Hiroaki Sakurai, Yann-Lii Leu, Shulhn-Der Wang, Hung-Yao Ho

**Affiliations:** 1 Graduate Institute of Biomedical Science, Chang Gung University, Guishan, Taoyuan Taiwan; 2 Department of Biomedical Sciences, College of Medicine, Chang Gung University, Guishan, Taoyuan Taiwan; 3 Healthy Aging Research Center, Chang Gung University, Guishan, Taoyuan Taiwan; 4 Metabolomics Core Laboratory, Chang Gung University, Guishan, Taoyuan Taiwan; 5 Clinical Phenome Center, Chang Gung Memorial Hospital at Linkou, Guishan, Taoyuan Taiwan; 6 Kidney Research Center, Chang Gung Memorial Hospital, Chang Gung University, School of Medicine, Taoyuan, Taiwan; 7 Department of Biochemistry, Chang Gung University, Guishan, Taoyuan Taiwan; 8 Research Center for Emerging Viral Infections, Chang Gung University, Guishan, Taoyuan Taiwan; 9 Molecular Infectious Disease Research Center, Chang Gung Memorial Hospital, Chang Gung University College of Medicine, Taoyuan, Taiwan; 10 Department of Pediatrics, National Cheng Kung University Hospital, College of Medicine, National Cheng Kung University, Tainan, Taiwan; 11 Department of Emergency Medicine, National Cheng Kung University Hospital, College of Medicine, National Cheng Kung University, Tainan, Taiwan; 12 Center of Infectious Disease and Signaling Research, National Cheng Kung University, Tainan, Taiwan; 13 Department of Cancer Cell Biology, Graduate School of Medicine and Pharmaceutical Sciences, University of Toyama, Toyama, Japan; 14 Graduate Institute of Natural Products, College of Medicine, Chang Gung University, Taoyuan, Taiwan; 15 Center for Traditional Chinese Medicine, Chang Gung Memorial Hospital at Linkou, Guishan, Taoyuan Taiwan; 16 Chinese Herbal Medicine Research Team, Healthy Aging Research Center, Chang Gung University, Taoyuan, Taiwan; 17 School of Post-Baccalaureate Chinese Medicine, College of Chinese Medicine, China Medical University, Taichung, Taiwan; 18 Department of Medical Biotechnology and Laboratory Science, College of Medicine, Chang Gung University, Taoyuan, Taiwan

## Abstract

No effective drug is currently available for treatment of enterovirus 71 (EV71) infection. *Schizonepeta tenuifolia* Briq. (ST) has been used as a herbal constituent of traditional Chinese medicine. We studied whether the aqueous extract of *Schizonepeta tenuifolia* Briq (STE) has antiviral activity. STE inhibited replication of EV71, as evident by its ability to diminish plaque formation and cytopathic effect induced by EV71, and to inhibit the synthesis of viral RNA and protein. Moreover, daily single-dose STE treatment significantly improved the survival of EV71-infected mice, and ameliorated the symptoms. Mechanistically, STE exerts multiple effects on enteroviral infection. Treatment with STE reduced viral attachment and entry; the cleavage of eukaryotic translation initiation factor 4 G (eIF4G) by EV71 protease, 2A^pro^; virus-induced reactive oxygen species (ROS) formation; and relocation of heterogeneous nuclear ribonucleoprotein A1 (hnRNP A1) from the nucleus to the cytoplasm. It was accompanied by a decline in EV71-associated hyperphosphorylation of p38 kinase and EPS15. It is plausible that STE may inhibit ROS-induced p38 kinase activation, and subsequent hnRNP A1 relocation and EPS15-mediated membrane trafficking in infected cells. These findings suggest that STE possesses anti-EV71 activities, and may serve as health food or candidate antiviral drug for protection against EV71.

## Introduction

Enterovirus 71 (EV71) is a non-enveloped, positive-sense single stranded RNA virus belonging to the family *Picornaviridae*, and is one of the major pathogens that causes hand, foot and mouth disease in young children, especially those under 5 years old^1^. EV71 transmits from person to person by direct contact saliva, nasal mucus, stool and blister of infected patients^2–4^. Most of EV71 infections are mild and self-limiting. These infected individuals have slight fever, herpangina, and rash on the mouth, hand and body. Some severe cases may progress to develop aseptic meningitis, encephalitis, and polio-like symptoms. The central nervous system (CNS) infections can result in pulmonary edema, pulmonary hemorrhage and lung collapse^5–8^, which may be fatal to patients. The survivors of severe EV71 infections may have irreversible neural sequelae^9^. Three large outbreaks of EV71 have been recorded in recent decades. In 1998, 129,106 individuals were infected, and 78 fatal cases were reported in Taiwan^1^. EV71 caused 126 deaths in 2008 and 567 victims in 2012 in China^10, 11^. No antiviral drug is available for treating EV71 infection. Development of a specific anti-EV71 drug may improve the clinical outcomes in infected patients.

The RNA genome of EV71 encodes a large precursor polypeptide that is processed by viral protease to generate viral structural proteins (VP1, VP2, VP3, and VP4) and nonstructural proteins (2A, 2B, 2C, 3A, 3B, 3C, and 3D). After viral attachment to receptor, the virus is endocytosed via clathrin-coated vesicles, and uncoated to release the genomic RNA. Unlike cellular message RNA, the viral genomic RNA lacks the cap structure on 5′ end to recruit ribosome and initiate protein translation. Its highly structured 5′ untranslated region (UTR) contains internal ribosomal entry site (IRES) which interacts with IRES *trans*-acting factors (ITAFs) and facilitates the initiation of translation^12, 13^. Heterogeneous nuclear ribonucleoprotein A1 (hnRNP A1) is one member of ITAFs and is involved in the synthesis of viral genome and protein^14, 15^. EV71 infection induces the relocation of hnRNP A1 from nucleus to cytoplasm which may be beneficial to viral replication^14^. The viral protease, 2A^pro^, hydrolyzes eukaryotic initiation factor 4G (eIF4G) and poly(A) binding protein (PABP) that are essential for the cap-dependent translation^16–18^. The hydrolytic reactions cause the switch of cap-dependent translation to IRES-dependent translation. EV71 hijacks the host translation machinery to synthesize viral proteins^18^.

Accumulating evidence indicates that the redox state of host may play important roles in the viral pathogenesis. The susceptibility to viruses, such as dengue virus, enterovirus 71 or coronavirus, is related to redox state of host cells^19–22^. Antioxidants are known to deter viral infection. For instance, glutathione and resveratrol inhibit influenza infection in cell and animal model^23, 24^. Treatment with synthetic antioxidants, such as mitoTEMPO and *N*-acetyl-cysteine, and natural antioxidants, such as epigallocatechin gallate (EGCG) and gallocatechin gallate (GCG), diminishes EV71 replication^20, 25, 26^. The antiviral capacity of tea polyphenols correlates with their antioxidant ability. These studies suggest that the redox state of host is an important determinant of viral susceptibility, and that the antioxidative capacity of herbal extract partly accounts for the antiviral activity.

*Schizonepeta tenuifolia* Briq. (ST), also called “Jing Jie” in China, is an annual plant belonging to the family Labiatae. In East Asia, the fresh stem and leaf of ST are usually used as ingredients in several food recipes, herbal tea, beneficial drinks, medicinal cuisine, and herbal remedy^27^. Spikes, stems and leaves of ST are sun-dried or carbonized before use medicinally. ST contains a number of bioactive constituents (Supplementary Table S1)^27–34^. ST is used to treat the common cold, headaches, fever, allergic dermatitis, skin rash, and inflammatory diseases^28^. An antiviral activity is associated with ST. ST has been used in formulation of yinqiao-decoction, which proved effective in reduction of hemagglutinin titer of virus in lung tissue of influenza virus-infected mice^35^. However, previous reports on the anti-enteroviral activity of ST extract are controversial. Hsu *et al*. showed that a 95% ethanolic extract of ST inhibits one out of four clinical isolated EV71 in a screen of anti-EV71 activity with Taiwanese folk medicinal plants. In contrast, Lin *et al*. showed that an aqueous extract of dried ST has no anti-EV71 activity among 22 antipyretic and toxin-eliminating traditional Chinese herbs^37^.

In the present study, we demonstrate an anti-EV71 activity of STE. STE inhibits cytopathic effect, plaque formation, and genomic RNA replication of EV71. Moreover, it alleviates the symptoms and enhances the survival of EV71-infected mice. Mechanistically, STE inhibits EV71-induced ROS generation and hnRNP A1 translocation; cleavage of eukaryotic translation initiation factor 4G (eIF4G); and p38 kinase-mediated EPS 15 phosphorylation. Our findings suggest that STE exerts an antiviral activity against EV71 through its action on a number of targets.

## Results

### STE inhibits EV71 replication

**Figure 1:**
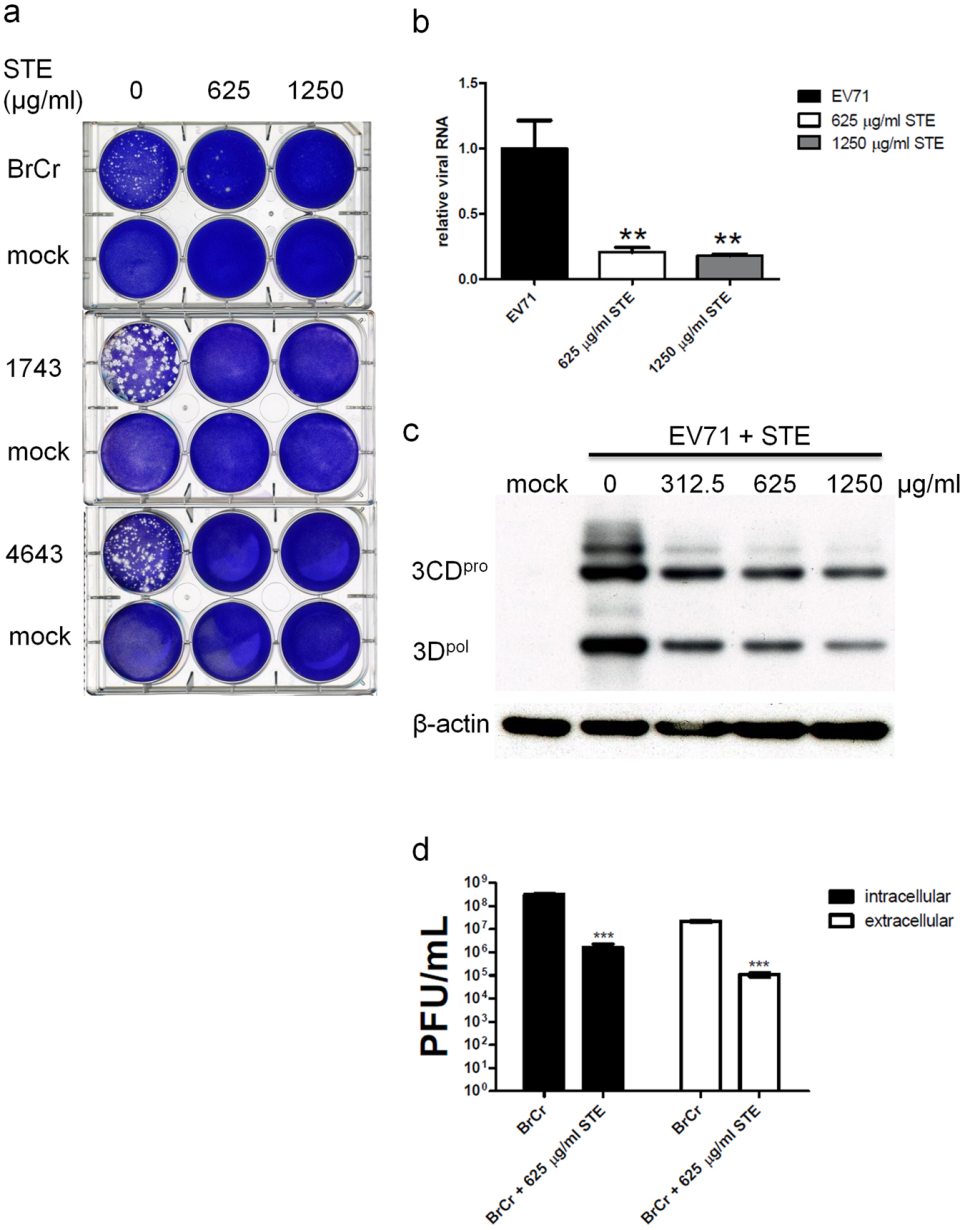
Anti-EV71 activity of STE. (**a**) RD cells were mock- or infected with 100 PFU of EV71 strains, namely BrCr, 1743 and 4643 for 1 h, and were overlaid with 0.3% agarose in DMEM/2% FBS, which was supplemented with 0, 625, or 1250 µg/ml of STE. After 96 h, the plates were fixed with 10% formalin, and stained with 1% crystal violet solution. Representative cell plates are shown here. (**b**) RD cells were infected with BrCr at m. o. i. of 0.05 in the absence or presence of 625 and 1250 µg/ml STE for 16 h. The level of EV71 genomic copy was determined by quantitative reverse transcription PCR, and normalized to the level of β-actin. Data are expressed relative to that of untreated cells. The results are means ± SD of three separate experiments. **P < 0.01, vs. infected cells without treatment. (**c**) RD cells were infected with BrCr at m. o. i. of 5 in the presence of indicated concentrations of STE. Cellular protein was harvested at 6 h p. i., and was subject to western blotting with antibodies to 3D and β-actin. The cropped images of the blots are shown. A representative experiment out of three is shown. (**d**) RD cells were infected with BrCr as described in (**c**), and treated without or with 625 µg/ml STE. Extracellular and intracellular viral particles were collected at 9 h p. i. for titer determination. The results are means ± SD of three separate experiments. ***P < 0.0001, vs. infected cells without treatment.

We used a modified plaque assay to screen for anti-enteroviral activity of herbal extract. RD cells were infected with 100 PFU of different EV71 strains such as BrCr, 1743 and 4643, and overlaid with agarose containing 0, 625, or 1250 µg/ml of STE. STE significantly reduced plaque formation by BrCr strain, and completely abrogated plaque formation by 1743 and 4643 strains on RD (Fig. 1a) and Vero cells (Supplementary Fig. S1b). At these concentrations, the STE did not have cytotoxic effect on RD cells (CC_50_ = 3903 μg/ml) (Supplementary Fig. S1a) and Vero cells (CC_50_ = 2268 μg/ml) (Supplementary Fig. S1a). To validate the antiviral effect of STE, we infected RD cells with BrCr strain at m. o. i. of 0.05, and quantified the level of genomic RNA. The level of EV71 RNA decreased by nearly 80% and 85% upon treatment with 625 and 1250 µg/ml STE, respectively (Fig. 1b). It was paralleled by a dose-dependent decrease in expression of EV71 proteins, including viral RNA dependent RNA polymerase (3D^pol^) and viral protease (3CD^pro^). The expression levels of 3D^pol^ were lowered by 67 and 80% in infected cells in the presence of 625 and 1250 µg/ml STE. Likewise, the expression levels of 3CD^pro^ decreased by 56 and 69% in infected cells treated with 625 and 1250 µg/ml of STE (Fig. 1c). Reduction in levels of genomic RNA and proteins of EV71 was accompanied by a significant decrease in progeny virus. The amounts of intracellular and extracellular viral particles decreased by more than 2 orders of magnitude in STE-treated infected cells, as compared to those of control treatment group (Fig. 1d). These findings demonstrate that STE possesses an anti-EV71 activity.

### STE inhibits EV71 infection at both attachment and post-attachment steps

**Figure 2:**
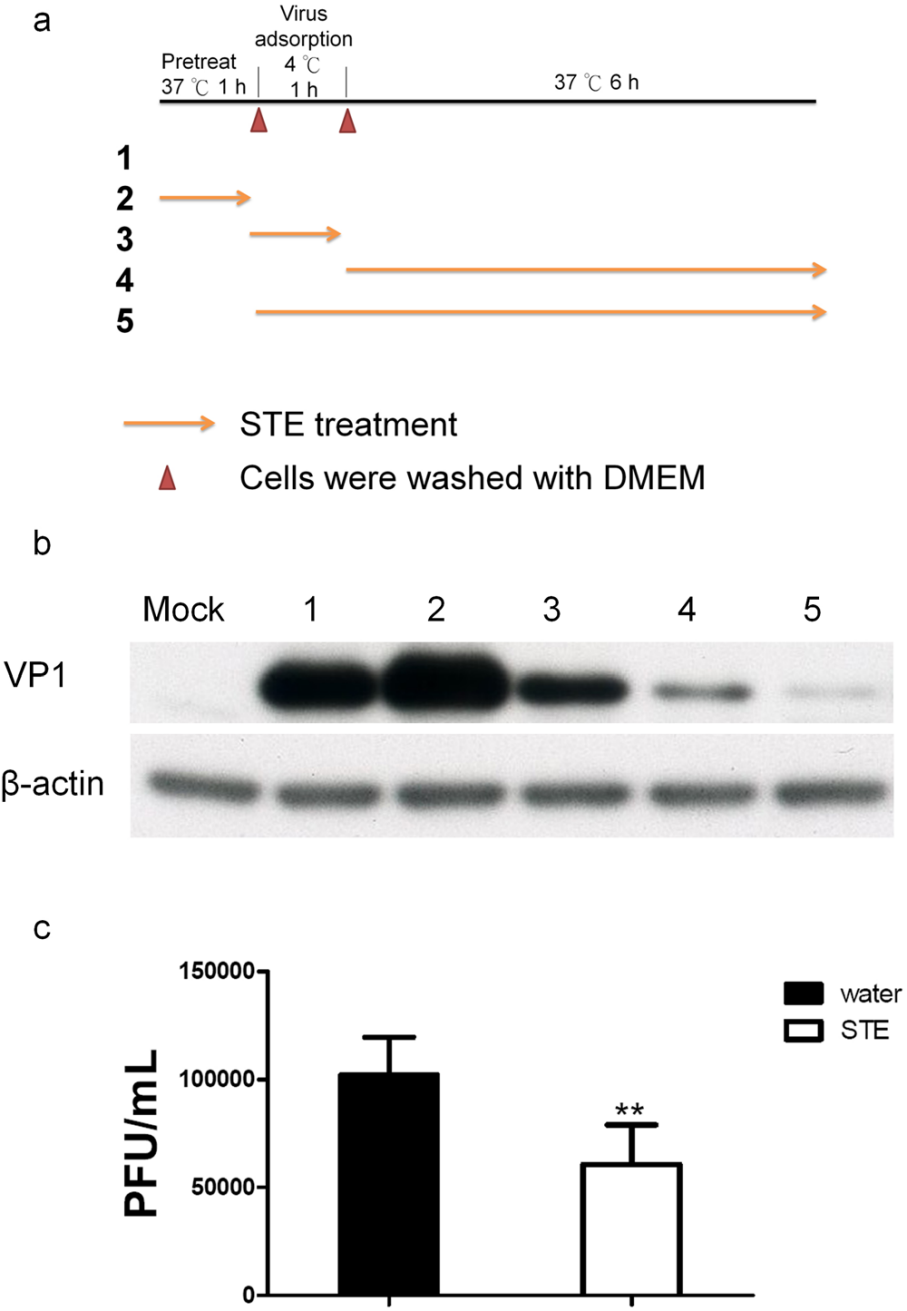
STE inhibits EV71 infection during adsorption and post-adsorption phases. (**a**) The time-of-addition experiment was performed to determine the stage of infection, at which STE exerts its antiviral activity. RD cells were mock-infected. RD cells were incubated with BrCr at m. o. i. of 20 at 4 °C, washed free of virus, and cultured for 6 h prior to analysis for VP1 expression (condition 1; positive control). Cells were pre-treated with 1250 µg/ml STE for 1 h, incubated with virus, washed, and cultured for 6 h before analysis (condition 2). Cells were incubated with virus in the presence of 1250 µg/ml STE for 1 h, washed, and cultured for 6 h before analysis (condition 3). Cells were incubated with virus, washed, and treated with STE for 6 h before analysis (condition 4). Cells were incubated with virus in the presence of 1250 µg/ml STE for 1 h, washed, and treated with STE for 6 h before analysis (condition 5). (**b**) Expression of VP1 and β-actin in cells treated under aforementioned conditions was determined by western blotting. The cropped images of the blots are shown. A representative experiment out of three is shown. (**c**) Interaction of virions with STE reduces the viral infectivity. The viral preparation was incubated with 1250 µg/ml STE or water at 4 °C for 1 h. The reaction mix was filtered through Amicon filter, and the retentate was assayed for viral titer. Data are means ± SD of three separate experiments. **P < 0.01, vs. water treatment group.

To study the mechanism of antiviral activity of STE, we performed the time-of-addition assay to study the stage at which STE inhibits EV71 infection. STE was added during different periods of BrCr infection (Fig. 2a), and immunoblotting to VP1 was conducted. Expression of VP1 is indicative of viral replication. As shown in Fig. 2b, treatment with STE prior to EV71 adsorption (condition 2) failed to suppress EV71 replication, and even slightly promoted it. STE treatment of cells during viral adsorption period (condition 3) moderately diminished EV71 replication. Addition of STE after viral adsorption period significantly suppressed EV71 replication (condition 4). If STE was present during viral-adsorption and post-adsorption periods (condition 5), the replication was severely repressed. These findings suggest that STE inhibits at both viral viral-adsorption and post-adsorption phases. Apparently, there was synergy between antiviral activities acting at these phases.

The antiviral activity of STE acting at viral adsorption period implies that STE may interact with virions making them unavailable for attachment to surface receptor. To test such possibility, we mixed the virus with STE or water; removed any excess STE using centrifugal filtration, and determined the titer of infectious virus. The titer of infectious virus in STE treatment group was 6.05 ± 1.84 × 10^4^ PFU/ml, which was significantly lower than that of water treatment group (1.02 ± 0.17 × 10^5^ PFU/ml) (Fig. 2c). These findings suggest that STE may interact with virions and interfere with their subsequent attachment to cellular receptor.

### STE hampers EV71-induced switch between cap-dependent and IRES-dependent translation in cells

**Figure 3:**
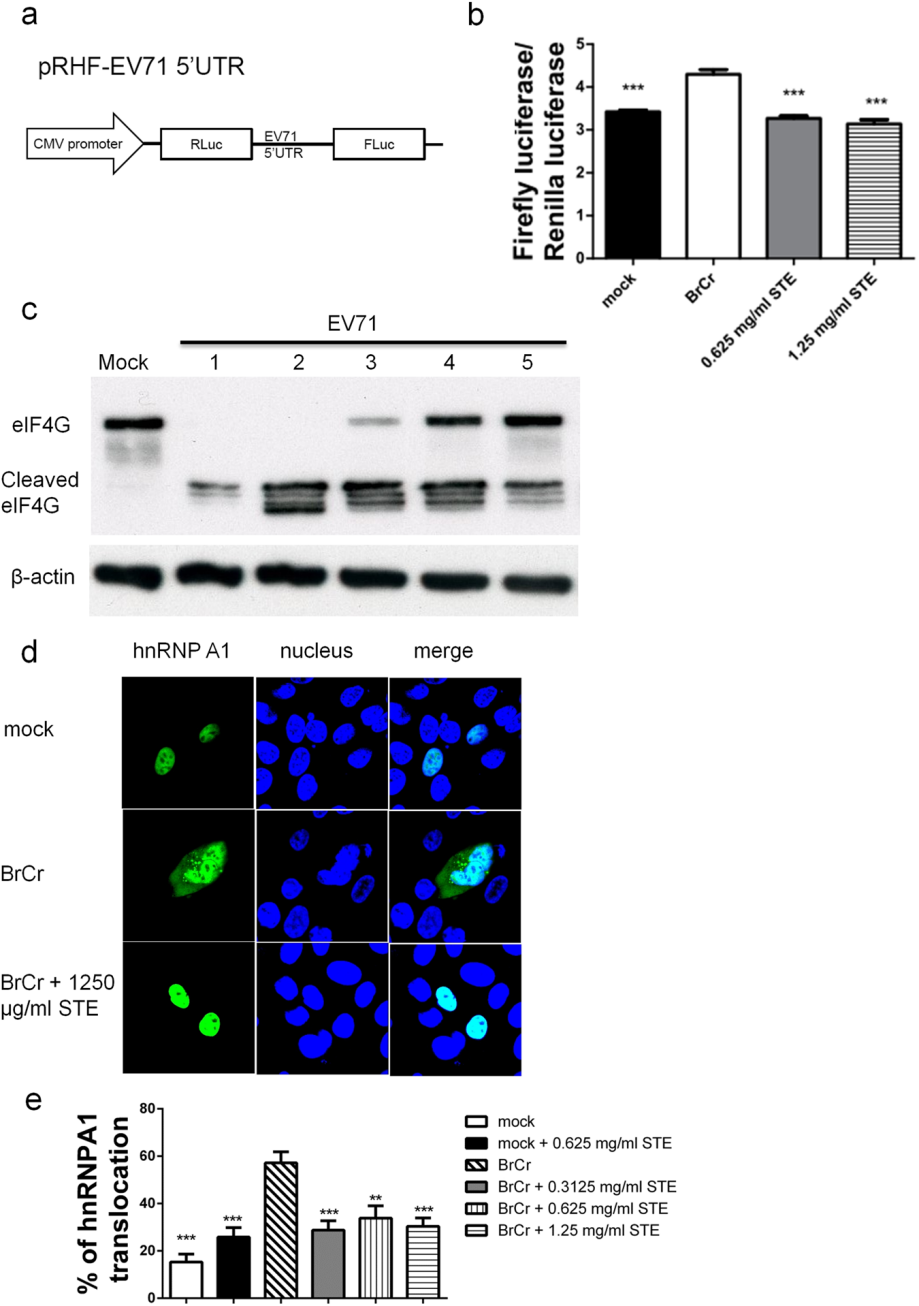
STE blocks EV71-induced shutoff of cap-dependent translation and initiation of IRES-dependent translation in host cells. (**a**) The bicistronic plasmid pRHF-EV71-5′UTR for analysis of cap-dependent and IRES-dependent translation is depicted. (**b**) RD cells were transfected with pRHF-EV71-5′UTR. Twelve hour later, the transfected cells were mock- or infected with BrCr at m. o. i. of 20 for 1 h, and treated with 625 or 1250 µg/ml STE for 6 h. The activities of firefly luciferase and renilla luciferase were measured. Data are means ± SD of three separate experiments. ***P < 0.0001, vs. infected cells without treatment. (**c**) RD cells were infected under conditions described in the legend of Fig. 2a. The number shown above the western blot image indicates the condition of treatment. Cells were harvested for western blotting with antibodies to eIF4G and β-actin. The positions of intact and cleaved eIF4G are indicated. The cropped images of the blots are shown. A representative experiment out of three is shown. (**d**) RD cells were transfected with expression vector encoding GFP-tagged hnRNP A1. Forty-eight hour later, the transfected cells were infected with EV71 at m. o. i. of 20 for 1 h, and subsequently treated with 1250 µg/ml STE for 6 h. The cells were fixed and stained with Hoechst 33342 for confocal microscopy. A representative experiment out of three is shown. (**e**) Cells were transfected, infected, and treated with indicated concentrations of STE as described in (**d**). The cells were fixed, stained with Hoechst 33342, and subject to image analysis using IN Cell Analyzer 1000. The percentage of cells showing cytoplasmic relocation of hnRNP A1 were quantified. Data are means ± SD of three separate experiments. **P < 0.005; ***P < 0.0001; vs. infected cells without treatment.

STE has antiviral activity on post-viral adsorption phase. The switch between cap-dependent and IRES-dependent translation occurs during the course of EV71 replication^13, 38–40^. To study whether STE affects this process, we transfected cells with a bicistronic reporter pRHF-EV71-5′UTR (Fig. 3a), infected the transfected cells for an hour, and subsequently treated them with STE. The firefly luciferase activity/Renilla luciferase activity (Fluc/Rluc) ratio of EV71-infected cells increased by 25%, as compared to that of uninfected cells (p < 0.0001). STE treatment in post-adsorption phase effectively inhibited such increase (Fig. 3b). The ratio of firefly and Renilla luciferase activities was indicative of the relative preponderance of IRES- and cap-dependent translation. These findings suggest that STE may abolish the EV71-induced switch from cap- to IRES-dependent translation. To further validate whether STE treatment prevents EV71-induced termination of host’s translation, we examined the level of translation initiation factor eIF4G, a target of enteroviral proteases. RD cells were infected with EV71 at m. o. i. of 20, and treated with STE under conditions depicted in Fig. 2. EV71 infection caused complete cleavage of eIF4G (condition 1). Pre-treatment with STE prior to viral adsorption (condition 2) did not affect this process. The presence of STE during viral adsorption phase partially blocked the cleavage (condition 3). Addition of STE to cells during post-adsorption significantly deterred eIF4G from cleavage (condition 4). Inclusion of STE during adsorption and post-adsorption phases synergistically inhibited eIF4G degradation (condition 5). These findings suggest that STE prevents the shut-down of cap-dependent translation through maintenance of active eIF4G.

It is plausible that STE interferes with IRES-dependent translation. It is known that a *trans*-acting factor hnRNP A1 relocates to cytoplasm, and interacts with EV71 5′ UTR to facilitate the translation^14^. To explore this possibility, we studied whether STE treatment affects the relocation of hnRNP A1 in infected cells. We transfected RD cells with expression vector encoding a GFP-tagged hnRNP A1; infected the transfected cells with BrCr, and studied the effect of STE treatment. As shown in Fig. 3d, the GFP-tagged hnRNP A1 was localized in nuclei of mock-transfected cells. EV71 infection caused relocation of hnRNP A1 from nucleus to cytoplasm. Treatment of infected transfectants with 1250 µg/ml STE inhibited such change in hnRNP A1 distribution. A high throughput imaging was applied to quantify the percentage of cells showing cytoplasmic relocation of GFP-tagged hnRNP A1. The percentage of such cells in mock-infected cells was 15.30 ± 3.33%, but it significantly increased to 57.15 ± 4.66% in EV71-infected cells. STE treatment caused a significant reduction in cytoplasmic GFP-positive cells. The percentage of cytoplasmic GFP-positive infected cells dropped to 28.76 ± 3.94% upon treatment with 312.5 µg/ml of STE (Fig. 3e). These findings suggest that STE may suppress IRES-dependent translation through inhibition of hnRNP A1 relocation.

### STE suppresses the EV71-induced activation of p38 kinase

**Figure 4:**
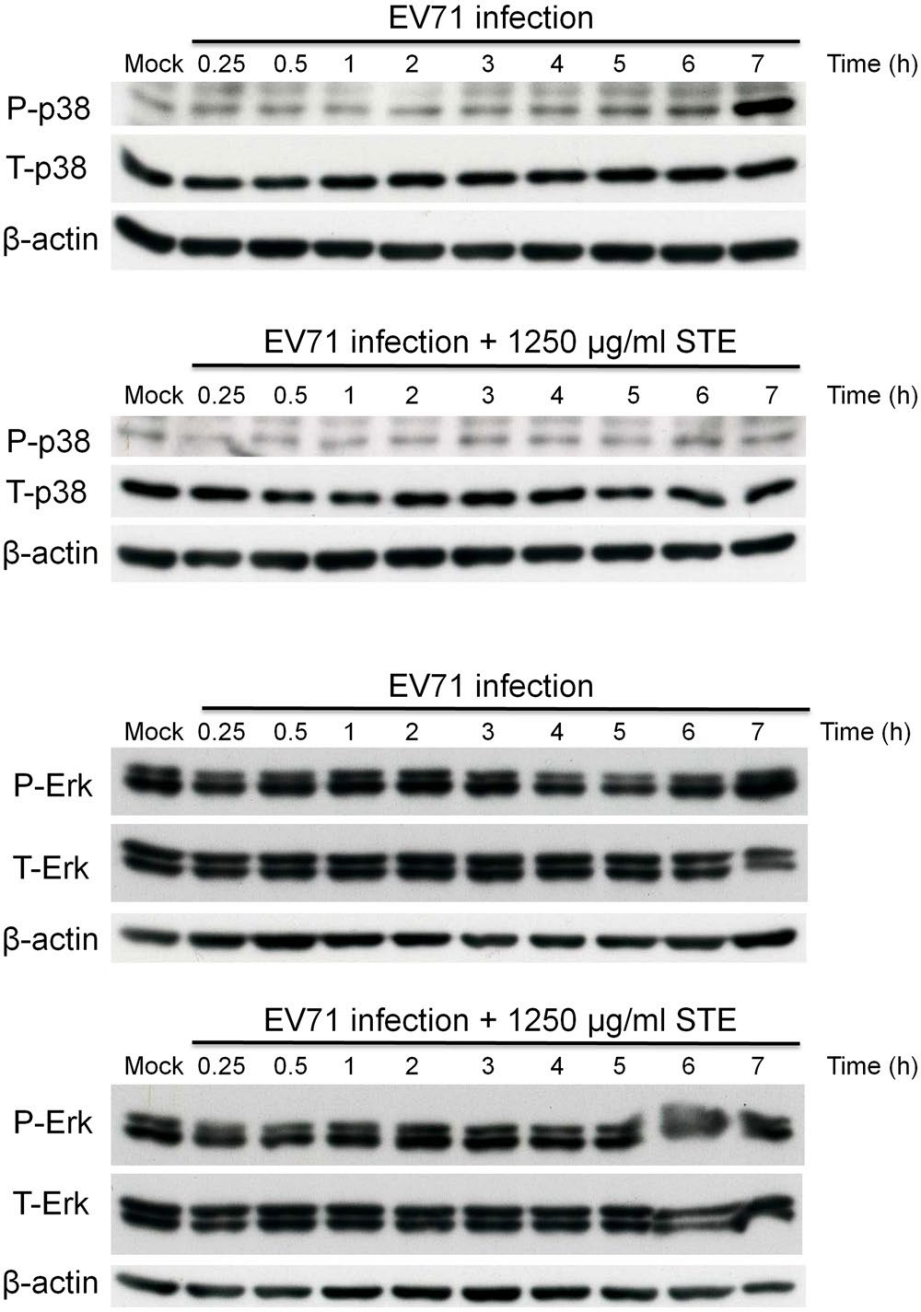
STE inhibits EV71-induced p38 kinase phosphorylation. RD cells were mock- or infected with BrCr at m. o. i. of 20 for 1 h, and subsequently treated with 1250 µg/ml STE. The treated cells were harvested at 0.25, 0.5, 1, 2, 3, 4, 5, 6, and 7 h p. i. Levels of p38 kinase and Erk1/2, and the corresponding phosphorylated forms (P-p38 and P-Erk) were examined by western blotting. The blot was stripped, and analyzed with antibody to β-actin. The cropped images of the blots are shown. A representative experiment out of three is shown.

**Figure 5:**
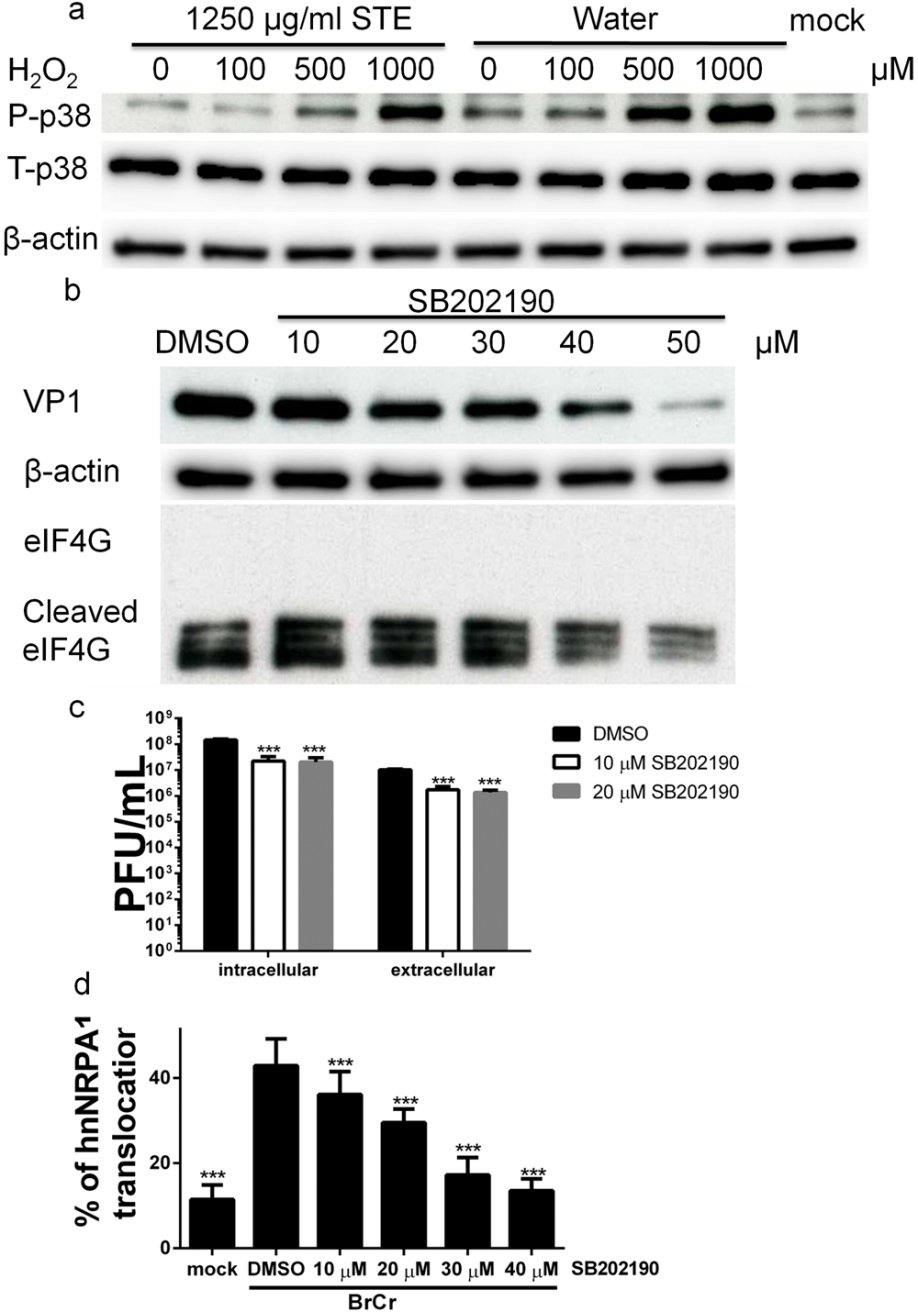
STE inhibits ROS-induced p38 kinase activation that is critical to EV71 infection. (**a**) RD cells were un- or treated with 100, 500 or 1000 μM H_2_O_2_ for 20 min in the absence or presence of 1250 μg/ml STE. Expression of total and phosphorylated p38 kinase and β-actin was examined by western blotting. The cropped images of the blots are shown. A representative experiment out of three is shown. (**b**) RD cells were pre-treated with DMSO or 10, 20, 30, 40, or 50 µM SB202190 for 1 h, and infected with BrCr at m. o. i. of 5 for 1 h. The cells were harvested at 9 h p. i. for western blotting with antibodies to VP1, eIF4G and β-actin. The cropped images of the blots are shown. A representative experiment out of three is shown. (**c**) Cells were treated with DMSO or SB202190, and infected as described in (**b**). Intracellular and extracellular progeny virus was collected at 9 h p. i. for titer determination. Data are means ± SD of three separate experiments. ***P < 0.0001, vs. infected cells with DMSO treatment. (**d**) RD cells were transfected with hnRNP A1-GFP. Twenty-four hour later, the transfected cells were pre-treated with DMSO or 10, 20, 30, 40, or 50 µM SB202190 for 1 h, and infected with BrCr at m. o. i. of 20 for 1 h. The cells were fixed at 6 h p. i., stained with Hoechst 33342, and subject to image analysis using IN Cell Analyzer 1000. The percentage of cells showing cytoplasmic relocation of hnRNP A1 were quantified. Data are means ± SD of three separate experiments. **P < 0.005; ***P < 0.0001, vs. infected cells with DMSO treatment.

The p38 kinase is known to regulate hnRNP A1 translocation^41^. It is possible that STE negatively regulates hnRNP A1 translocation through its modulation of p38 signaling. To test such possibility, we infected RD cells with BrCr strain at m. o. i. of 20, treated the infected cells without or with 1250 µg/ml of STE, and harvested them for immunoblotting to phosphorylated p38 kinase and Erk. EV71 infection appeared to cause biphasic activation of p38 kinase. There was a modest increase in p38 kinase phosphorylation at around 15–30 min p. i. A second phase of stronger phosphorylation occurred at 3 h p. i., and the intensity of p38 kinase phosphorylation steadily increased up to 7 h p. i. (Fig. 4). STE treatment significantly inhibited both phases of p38 kinase phosphorylation (Fig. 4). On the contrary, EV71 infection did not induce phosphorylation of Erk 1 and 2 until at 7 h after viral adsorption. STE treatment did not significantly alter the kinetics of Erk phosphorylation. The essential role of p38 kinase activation in EV71 infection was demonstrated by the inhibitory effect of p38 kinase inhibitor SB202190 on VP1 expression (Fig. 5b, middle panel). The level of VP1 in EV71-infected cells was reduced by SB202190 in a dose-dependent manner. Moreover, the production of progeny virus was suppressed by SB202190. The levels of intracellular and extracellular progeny virus produced by cells, which were treated with 10 or 20 µM SB202190, were over 85% lower than that of DMSO-treated cells (Fig. 5c). These findings suggest that EV71 infection specifically activates p38 kinase, and STE suppresses p38 kinase activation. Intriguingly, treatment with SB202190 did not rescue eIF4G from cleavage (Fig. 5b, middle panel), implying that the EV71-induced eIF4G cleavage is independent of p38 kinase activation.

To study the possibility that p38 kinase pathway mediates cytoplasmic relocation of hnRNP A1, we examined the effect of SB202190 on hnRNP A1 distribution in infected cells. As shown in Fig. 5d, the percentage of cytoplasmic relocation of hnRNP A1 in BrCr-infected cells decreased in a dose-dependent manner. Such finding suggested that EV71 activates cytoplasmic hnRNP A1 translocation via p38 kinase pathway.

### STE reduces p38 kinase phosphorylation through an antioxidative mechanism

**Figure 6:**
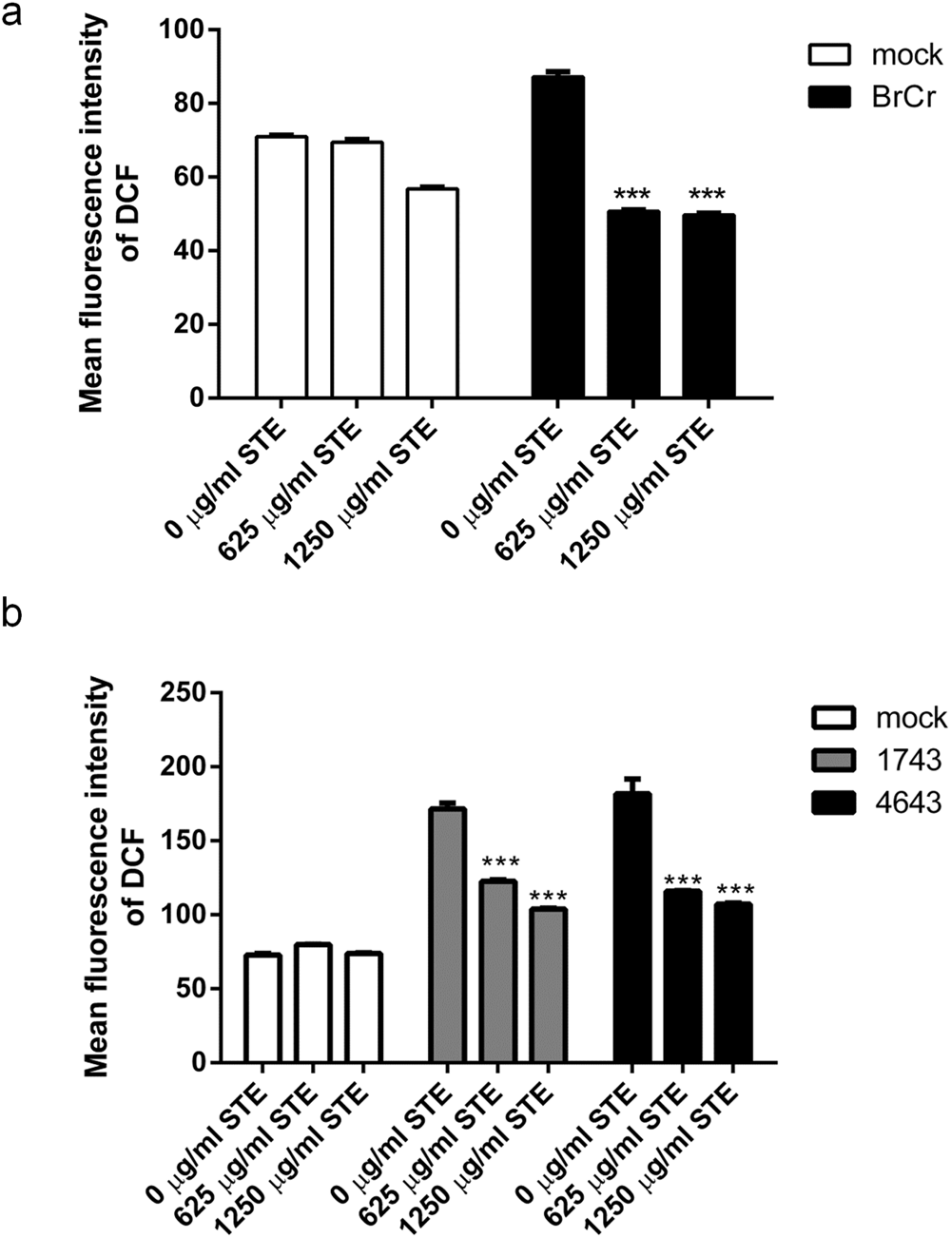
STE suppresses EV71-induced ROS generation. RD cells were mock- or infected with BrCr, 1743, and 4643 at m. o. i. of 0.05 for 1 hr, and subsequently treated with indicated concentrations of STE for 24 h. Cells were loaded with H_2_DCFDA at 37 °C for 30 min, and analyzed cytometrically. Data are means ± SD of six separate experiments. ***P < 0.0001, vs. infected cells without treatment.

It has been known that hydrogen peroxide can induce p38 kinase phosphorylation^42, 43^, and that EV71 infection is associated with increased oxidative stress^25, 44^. This raises the possibility that EV71 may activate p38 kinase through ROS signaling, and STE may inhibit ROS signaling. To study such hypothesis, we first examined the antioxidative activity of STE. The antioxidative capacity of STE was quantified using the ferric reducing antioxidant power (FRAP) assay, and was 105.9 mg of Trolox equivalent per gram of lyophilized STE. To further study the antioxidative roles of STE in curbing EV71 infection-associated ROS generation, we stained EV71-infected cells with fluorescent probes, H_2_DCFDA or CellROX Deep Red reagent, to determine ROS generation in EV71-infected cells. Elevated ROS levels were detected in BrCr-, 1743-, and 4643-infected cells, as compared with that of mock-infected cells (Fig. 6 and Supplementary Fig. S2). However, STE caused significant reduction in ROS generation.

We proceeded to study whether STE affects activation of p38 kinase. We treated RD cells with H_2_O_2_, and examined the effect of STE on phosphorylation of p38 kinase. Hydrogen peroxide treatment caused a dose-dependent increase in p38 kinase phosphorylation in RD cells (Fig. 5a; lane 5–8). Treatment with STE significantly reduced both basal level of (lane 1 vs. lane 5) and H_2_O_2_-induced increase (lane 2–4 vs. lane 6–8) in phosphorylation. These findings suggest that ROS-induced p38 kinase activation can be inhibited by STE.

### EV71 infection induces phosphorylation of epidermal growth factor receptor substrate 15 (EPS15)

**Figure 7:**
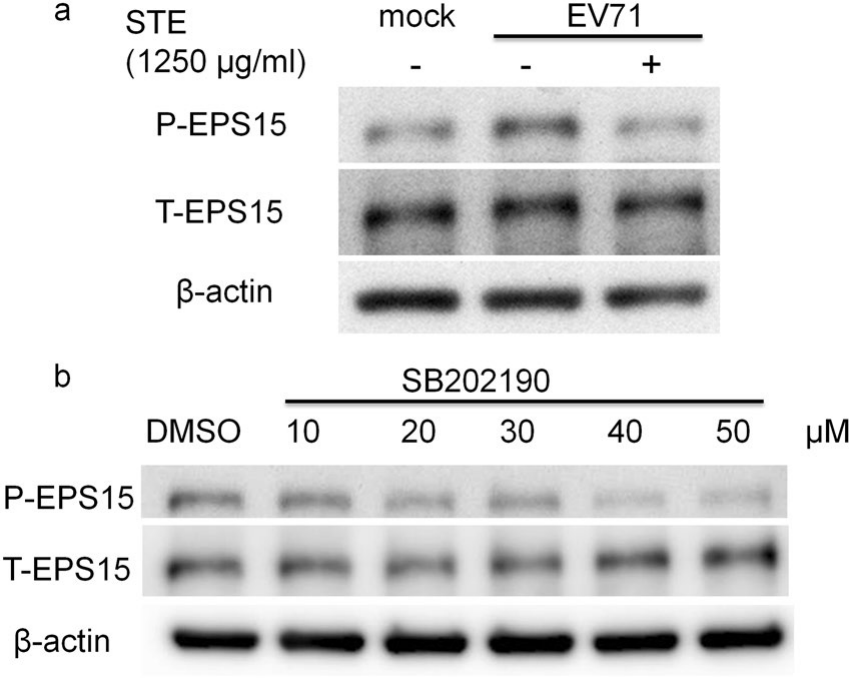
STE reduces EV71-induced phosphorylation of EPS15 at Ser796. (**a**) RD cells were mock- or infected with BrCr at m. o. i. of 20 for 1 h, and treated without or with 1250 µg/ml STE. Cells were harvested at 7 h p. i. for western blotting with antibodies to total EPS15 and the form phosphorylated at Ser796. The cropped images of the blots are shown. A representative experiment out of three is shown. (**b**) RD cells were pre-treated with DMSO or 10, 20, 30, 40, or 50 µM SB202190 for 1 h, and infected with BrCr at m. o. i. of 5 for 1 h. The cells were harvested at 9 h p. i. for western blotting with antibodies to phosphorylated and total EPS15. The cropped images of the blots are shown. A representative experiment out of three is shown.

Another target protein of p38 kinase, EPS15, is implicated in membrane trafficking^45, 46^, which may play important roles in RNA synthesis and virus assembly^47^. EV71 infection in RD cells induced EPS15 phosphorylation at Ser796 (Fig. 7a, lane 1 vs. 2). Treatment of EV71-infected cells with STE significantly reduced phosphorylation of EPS15 (Fig. 7a, lane 2 and 3). To validate whether p38 kinase pathway mediates EPS15 phosphorylation, we studied the effect of p38 kinase inhibitor SB202190 on EPS15 phosphorylation in EV71-infected cells. Phosphorylation of EPS15 in EV71-infected cells was reduced in the presence of SB202190 (Fig. 7b). These findings suggest that p38 kinase is involved in EPS15 phosphorylation. It is probable that STE inhibits EPS15 phosphorylation via its suppressive effect on p38 kinase.

### STE treatment promotes the survival rate and alleviates the neurological symptoms of EV71-infected mice

**Figure 8:**
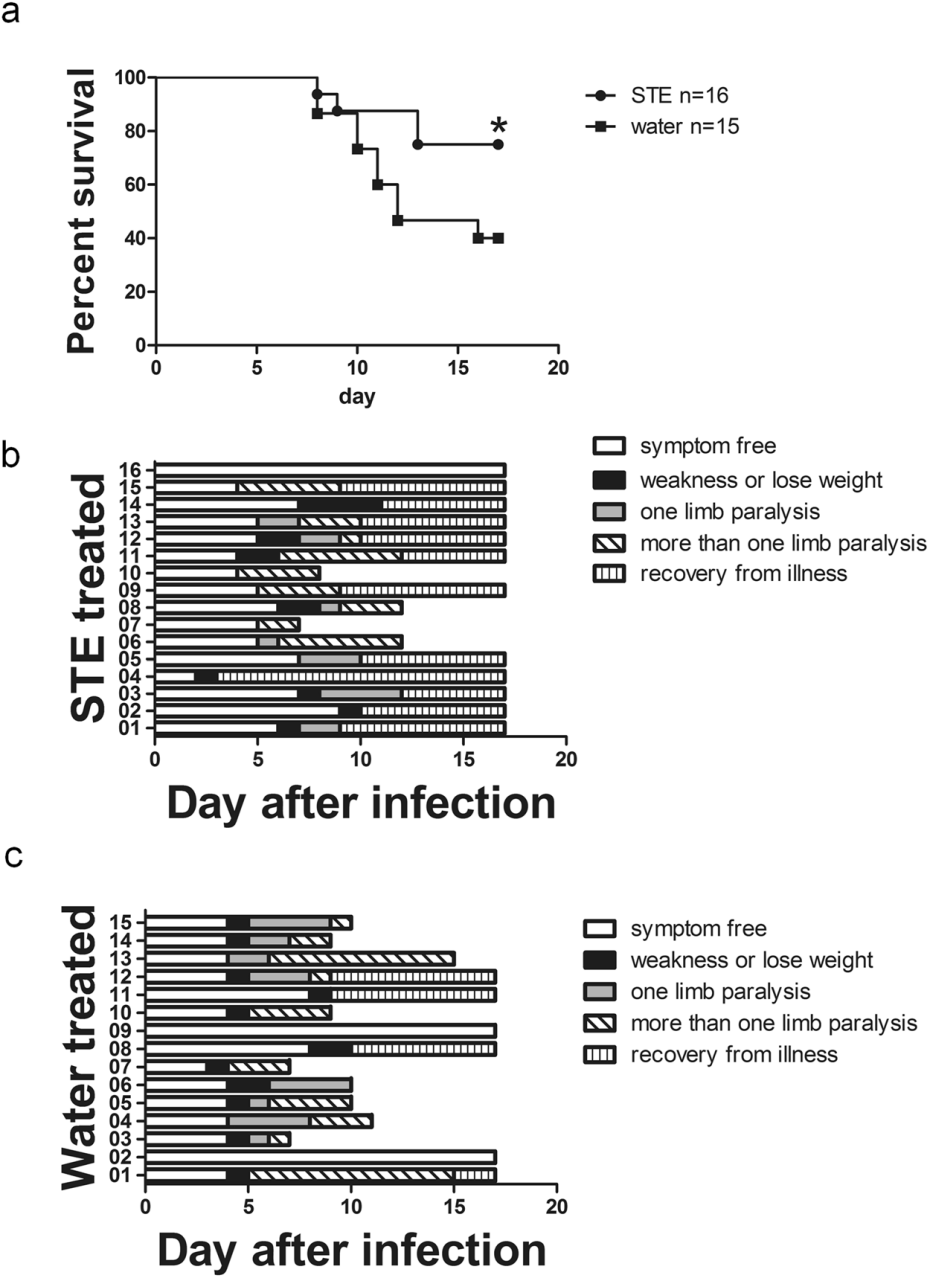
STE alleviates the clinical symptoms of and enhances the survival of EV71 infected mice. (**a**) Seven-day-old ICR mice were intraperitoneally infected with 2 × 10^6^ PFU MP4. One day later, EV71-infected mice were treated with single dose of STE (250 mg/kg) (n = 16) or water (10 ml/kg) (n = 15) daily for 14 days. The survival rate (**a**), and clinical symptoms in STE-treated (**b**) or water-treated (**c**) mice were recorded daily for 17 days. The clinical symptoms, including weakness, weight loss, paralysis of one limb, paralysis of more than one limb, and death, were recorded daily.

To study if STE treatment protects mice from EV71 infection, we infected mice with EV71, treated them with STE (250 mg/kg), and examined the survival and clinical signs. The survival rate of STE-treated infected mice (75%, n = 16) was significantly higher than that of water-treated infected mice (40%, n = 15) (Fig. 8a). The STE-treated infected mice displayed milder symptoms and rehabilitated more quickly than their water-treated counterparts. A higher percentage of infected mice in water treatment group showed paralysis of more than one limb than those in STE treatment group (Fig. 8b and c). Additionally, most of severely paralyzed mice in water treatment group did not recover from illness. These findings suggest that STE offers protection against EV71 infection *in vivo*.

## Discussion

The present study has shown that STE offers protection against EV71 *in vitro* and *in vivo*. Mechanistically, it exerts antiviral activities via a number of different mechanisms. It interferes with viral attachment to host cells; suppresses the cleavage of eIF4G; and hinders the cytoplasmic translocation of hnRNP A1. Cytoplasmic relocation of hnRNP A1 involves ROS signaling and p38 kinase activation. STE can act to scavenge ROS and to inhibit p38 kinase phosphorylation. In addition, another target molecule of p38 kinase, EPS15, is phosphorylated in EV71-infected cells. EPS15 phosphorylation is inhibited by STE. Our findings suggest that STE exerts powerful antiviral activity against EV71.

There has not been effective drug available for treatment of EV71 patients. Controversial results about the antiviral effect of STE have been reported^36, 37^. Using a modified plaque assay, we have screened herbal extracts for their anti-EV71 activities, and found that STE offered protection against BrCr, 1743, and 4643 strains. The STE inhibited CPE, and reduced levels of genomic RNA of and plaques formed by progeny virus. Difference in the reported *in vitro* antiviral activity of STE may be attributed to the difference in the methods of extraction. Hsu *et al*. used a combination of organic solvents for extraction^36^, while Lin *et al*. used boiling water for the same purpose^37^. Difference in extraction methods may affect the nature and amount of pharmacologically active constituents extracted^48^. Additionally, STE alleviates the clinical symptoms, and enhances the survival of EV71-infected mice. Our findings indicate that STE has antiviral activity against EV71 both *in vitro* and *in vivo*.

The mechanistic steps at which STE inhibits EV71 infection has been determined using time-of-addition assay. STE acts at both viral adsorption and post-adsorption stages. Treatment of virus with STE reduced the amount of infectious virus (Fig. 2c). Apparently, STE directly interacts with virions, making them unavailable for infection. It is probable that some constituents of STE bind to virion, and interferes with attachment of virion with receptor. It has been known that triterpenoids from *Glycyrrhiza uralensis* and *Ganoderma lucidum* can block adsorption and uncoating of enterovirus^49, 50^. Though triterpenoids are not major constituents of *Schizonepeta tenuifolia* Briq, it is possible that some natural compounds present in STE may act in a similar manner. Alternatively, STE may directly inactivate virion.

A number of mechanisms account for the ability of STE to inhibit infection at post-adsorption stage. STE blocks the EV71-induced suppression of host cell translation and the switch to viral translation. Viral protease 2A of EV71 can cleave eIF4G and PABP^16, 17^, both of which are necessary for translation of host cell mRNA. It is possible that STE directly inhibits protease 2A. Rosmarinic acid, one of bioactive constituents of STE^29^, is known to inhibit cysteine protease^51^. Another possible but not exclusive explanation is that STE reduces translation of viral RNA and thus the level of protease 2A. Initiation of viral RNA translation entails binding of ITAFs and host initiation factors to type I IRES element on 5′UTR^12^. One member of ITAFs, hnRNP A1, interacts with stem loop II of IRES, which is required for enteroviral translation and replication^14, 15^. EV71 infection induces translocation of hnRNP A1 from nucleus to cytoplasm (Fig. 3d), where it stimulates IRES activity^13^. A similar observation has been made in poliovirus-infected cells^52^. The ability of STE to suppress cytoplasmic relocation of hnRNP A1 may accounts for reduced enteroviral translation and replication. Besides, apigenin has been shown to prevent interaction between EV71 RNA and hnRNP A1^53, 54^. The glycosidic derivative of apigenin, apigenin-7-*O*-β-D-glucopyranoside, is also a constituent of STE^29^. It is currently not clear if the glycosidic derivative functions in the same way as apigenin.

The shuttling of hnRNP A1 between cytoplasm and nucleus can be regulated by ROS. Arsenite treatment that induces oxidative stress causes cytoplasmic relocation of hnRNP A1^55–57^. We have also found that hydrogen peroxide treatment of RD cells results in translocation of hnRNP A1 from nucleus to cytoplasm (Supplementary Fig. S3). It has been shown that EV71 infection causes mitochondrial dysfunction and NADPH oxidase activation^25, 44^, resulting in ROS generation. The increase in ROS may elicit subsequent signaling and hnRNP A1 relocation. STE may act as ROS scavenger to block hnRNP A1 translocation. Consistent with such notion, STE displays strong antioxidant capacity^27^. Moreover, STE significantly reduced ROS generation in EV71-infected cells (Fig. 6 and Supplementary Fig. S2). It is possible that ROS scavenging by STE diminishes hnRNP A1 translocation.

EV71-induced signaling may involve participation of MAPK-related pathways. Activation of p38 kinase promotes EV71 replication^58, 59^. Inhibition of p38 kinase suppressed enteroviral translation (Fig. 5b). It is likely that p38 kinase play important roles in EV71 infection. Consistent with this, the p38 kinase signaling cascade is involved in regulation of the subcellular distribution of hnRNP A1^58^. Activation of p38 kinase is sufficient to promote relocation of hnRNP A1^41^. Pharmacological inhibition of p38 kinase blocked such translocation process (Fig. 5d). We have shown that STE inhibited EV71-induced activation of p38 kinase (Fig. 4). The mechanism underlying the action of STE remains elusive. It has been known that ROS themselves are activator of p38 kinase^60–62^. Hydrogen peroxide treatment caused p38 kinase phosphorylation, which was suppressed in the presence of STE (Fig. 5a). Antioxidant treatment inhibits p38 kinase activation^60, 62^. It is possible that STE scavenges ROS to reduce activation of p38 kinase. In addition, STE may affect expression of enzymes involved in redox metabolism. For instance, STE down-regulates expression of inducible nitric oxide synthase^63^. Oral administration of STE induces hepatic expression of catalase, superoxide dismutase and glutathione peroxidase in a mouse model of inflammation^27^. These findings suggest that STE may suppress activation of p38 kinase and hnRNP A1 relocation via an antioxidative mechanism. EPS15, another target of p38 kinase, appears to play important roles in EV71 infection. It was identified independently as a phosphorylated protein of EGFR complex in EGF-stimulated cells^64^, and as a binding protein of α-adaptin and oncogenic variant of Crk^65, 66^. Confocal microscopic study revealed co-localization of EPS15, α-adaptin and clathrin in clathrin-coated pits^67^. Silencing of EPS15 expression reduces the endocytosis of EGFR and transferrin moderately^68^. These findings imply that EPS15 is involved in ligand-induced endocytosis^69^. Recent findings have suggested that phosphorylation of EPS15 modulates its biochemical activity^46^. Phosphorylation of Tyr850 of EPS15 is required for ligand-induced endocytosis^69^. EPS15 is phosphorylated at Ser796 by p38 kinase, and the location of this phosphorylation site implies a functional role in endocytic process^70^. It has been revealed that EPS15 plays an essential role in EV71 infection. Knockdown of EPS15 significantly reduces the extent of enteroviral infection^71^. Such result has been interpreted as the essentiality of endocytosis in infectious entry process of EV71. What is more, EPS15 may be implicated in membrane trafficking essential to EV71 replication. We found a robust increase in phosphorylation of p38 kinase and EPS15 during the mid- to late phase of EV71 life cycle (Figs 4 and 7), suggesting a role for EPS15 in replication. In agreement with this, enterovirus exploits the endocytic machinery to transfer cholesterol from plasma membrane and culture medium to replication organelles, where cholesterol facilitates RNA replication and 3CD^pro^ processing^47^. The antiviral activity of STE may be, at least in part, accounted for by its ability to inhibit p38 kinase and EPS15 phosphorylation.

Based on our findings, we propose a model for antiviral activity of STE (Supplementary Fig. S4). In this scheme, STE can act in a number of ways to deter viral infection. STE binds to virions to suppress their adsorption and internalization; inhibits cleavage of eIF4G; scavenges ROS and suppresses p38 kinase activation, quelling cytoplasmic relocation of hnRNP A1 and post-translational modification of EPS15. STE contains various bioactive constituents, some of which may have specific antiviral activities mentioned above.

## Methods

### Chemical drugs

Unless otherwise stated, all chemicals were purchased from Sigma-Aldrich (St. Louis, MO, USA). Dulbecco’s modified Eagle’s medium (DMEM), modified Eagle’s medium (MEM), fetal bovine serum (FBS), antibiotics, trypsin-EDTA, non-essential amino acid (NEAA), glutamine, and trypan blue were acquired from Thermo Fisher Scientific Inc. (Waltham, MA, USA). The fluorogenic dyes, CellROX deep red reagents and 2′,7′-dichlorodihydrofluorescein diacetate (H_2_DCFDA), were purchased from Thermo Fisher Scientific Inc. (Waltham, MA, USA).

### Cell culture and virus preparation

African green monkey kidney epithelial cells (Vero; ATCC CCL-81) were cultured in MEM with 10% heat-inactivated FBS, 100 U/ml penicillin, 0.1 mg/ml streptomycin, and 2.5 μg/ml amphotericin. Human rhabdomyosarcoma cells (RD; ATCC CCL-136) were maintained in DMEM supplemented with 10% FBS, 100 units/ml penicillin, 0.1 mg/ml streptomycin, 2.5 μg/ml amphotericin, 2 mM L-glutamine, and 0.1 mM NEAA. Cells were cultured at 37 °C in a humidified environment of 5% CO_2_. Three clinical isolated EV71 strains were used in this study. The prototype strain of EV71, CA-BrCr-70 (BrCr), belongs to genogroup A. Clinical isolate TW-1743-98 (1743) belongs to genogroup B4, while Tainan/4643/TW (4643) is classified in genogroup C2. Virus stocks were propagated in Vero or RD cells as previously described^20^. The mouse adapted EV71 strain, MP4, was produced from an infectious clone, MP4/y5. The infectious clone plasmid was linearized with *Mlu*I. *In vitro* transcription was performed using the MEGAscript T7 Transcription Kit (Thermo Fisher Scientific, Waltham, MA, USA). RD cells were set in 6-well plates at 4 × 10^5^ per well and incubated overnight. Three microgram of viral RNA was transfected into RD cell using lipofectamine 2000 (Thermo Fisher Scientific, Waltham, MA, USA) according to the manufacturer’s instructions. After 24 h incubation, the virus particles were harvested in three freeze-thaw cycles. The MP4 virus was further propagated in RD cells once before animal study. About 7.2 × 10^6^ RD cells were seeded into 15 cm culture dish and incubated at 37 °C in a 5% CO_2_ incubator overnight. The plated cells were washed with PBS twice, and infected with MP4 in DMEM supplemented with 2% FBS. After 48 h of infection, the virus supernatant was harvested^72^.

### STE preparation

STE was supplied and authenticated by Sun Ten Pharmaceutical Co. Ltd., (Taipei, Taiwan). A voucher specimen (CGU-ST-01) was deposited in the herbarium of Chang Gung University, Taoyuan, Taiwan. Five gram of lyophilized powder was dissolved in 50 ml distilled water with constant shaking at room temperature over a period of 16 h. Any insoluble substance was removed by centrifugation at 428 × g for 15 min and subsequent centrifugation at 27000 × g for 30 min at 4 °C. The aqueous extract of ST was filtrated through a 0.22 μm filter. The drug was diluted with culture medium before use and stored at 4 °C.

### Viral plaque assay and modified plaque assay screening for antiviral activity

The titer of virus was determined by plaque assays with Vero or RD cells. The viral supernatant was serially diluted with serum free medium in a ten-fold manner. The monolayer cells at a confluence of 80% were infected with diluted viral supernatants, and the viral titer was quantified in the form of plaque forming unit (PFU) per ml as previously described^26^.

For plaque reduction assay, cells in six-well plates were infected with 100 PFU of virus for 1 h at 37 °C. After removal of unabsorbed virus, cells were overlaid with 0.3% agarose in medium supplemented with 2% serum and different concentrations of STE. In the control group, mock infected cells were treated with the same concentrations of STE. The plaque size and number were determined for assessment of the antiviral activity of STE.

### Determination of genomic copy number of EV71

The monolayer of RD cells was infected with EV71 at multiplicity of infection (m. o. i.) of 0.05 in serum free medium. After 1 h viral absorption, DMEM supplemented with 2% FBS and different concentrations of STE was added to infected cells. The total RNA of infected cells at 16 hour post infection (p. i.) was extracted using total RNA mini Kit (Geneaid, Sijhih District, New Taipei City, Taiwan) according to manufacturer’s instructions. The relative copy number of EV71 was quantified as previously described^26^.

### SDS-PAGE and western blot analysis

Protein samples from cells were subject to by SDS-PAGE and western blot analysis as previously described^25^. Antibodies used in this study are listed in Supplementary Table S2.

### Infectivity reduction assay

EV71 (BrCr) was diluted to 1 × 10^5^ PFU/ml in 10 ml, and incubated with or without 1.25 mg/ml STE for 1 h on ice. The medium containing unadsorbed virus was transferred to Amicon Ultra centrifugal filter unit with Ultracel-100 membrane (#UFC910024, Merck Millipore Darmstadt, Germany), and STE was removed by centrifugation at 5000 × g at 4 °C for 30 min. Viral particles in the retentate were resuspended in 1 ml serum-free DMEM. The titer of virus was quantified by plaque assay.

### Ferric Reducing/Antioxidant Power (FRAP) Assay

The antioxidant power of STE was measured through the formation of ferrous tripyridyltriazine as previously described^73^. The method is based on the ability of antioxidant to reduce ferric-tripyridyltriazine complex to the ferrous form, which has an absorption maximum at 593 nm.

### Detection of Cellular Reactive Oxygen Species (ROS)

For determination of the intracellular ROS formation, cells were stained with cell-permeable fluorogenic dyes, CellROX Deep Red reagent or H_2_DCFDA as previously described^20, 25^. The method is based on the ability of intracellular ROS to oxidize the leuco dye to its fluorescent form, which makes dye-laden cells amenable to cytometric analysis.

### Bicistronic reporter assays for detection of IRES activity

Bicistronic plasmid pRHF-EV71-5′UTR was a gift from Dr. Shin-Ru Shih, Chang Gang University. This plasmid was constructed as previously described^74^, and encodes bicistronic mRNA in which expression of *Renilla* luciferase (RLuc) is controlled under cytomegalovirus (CMV) promoter, and expression of firefly luciferase (FLuc) is regulated by IRES of EV71. The plasmid DNA (0.5 µg) was transfected into RD cells with 1.5 µl of Lipofectamine 2000 according to manufacturer’s protocols. Twelve hours after transfection, cells were un- or infected with 6 × 10^6^ PFU EV71 for an hour. One hour later, DMEM/2% FBS supplemented with indicated final concentrations of STE was added to each well. After 6 h, cell lysates were harvested and enzyme activities were measured with dual-luciferase reporter assay system (Promega, Madison, USA) and GloMax 20/20 single tube luminometer (Promega, Madison, USA) according to manufacturer’s instructions.

### Heterogeneous nuclear ribonucleoprotein A1 (hnRNP A1) translocation assay

The plasmid pGFP-hnRNP A1 encodes a green fluorescent protein (GFP) tagged hnRNP A1 protein. RD cells were transfected with 0.5 µg of pGFP-hnRNP A1 plasmid using Lipofectamine 2000. At 48 h post-transfection, the cells were infected with or without 6 × 10^6^ PFU BrCr for 1 h at 37 °C. After virus adsorption, DMEM containing 2% FBS and different concentrations of STE was added, and the infected cells were incubated for another 6 h at 37 °C. Infected and mock treated cells were fixed with 10% formalin for 30 min, and the cell nuclei were stained with Hoechst 33342 (Thermo Fisher Scientific Inc., Waltham, MA, USA) in PBS at room temperature. The images for the fluorescence assay were obtained using IN Cell Analyzer 1000 (GE Healthcare Life Sciences, USA). The region of nucleus was defined as a blue fluorescent region. The translocation of hnRNP A1 from nucleus to cytoplasm is visualized as an increase in the intensity of green fluorescence in cytoplasm over that in nucleus. The percentage of cells showing hnRNP A1 relocation was quantified from 20 random fields per well.

For confocal microscopic study, RD cells were seeded in 35 mm glass bottom culture dish coated with poly-D-lysine (MatTek Corporation, MA, USA), and treated as described above. The stained cells were examined with Zeiss LSM 780 system (Carl Zeiss Microimaging GmbH, Heidelberg, Germany) as previously described^26^.

### Animal Study

The specific-pathogen-free, seven-day-old ICR mice (Laboratory Animal Center, College of Medicine, National Cheng Kung University, Tainan, Taiwan) were infected intraperitoneally with 2 × 10^6^ PFU MP4 suspended in 10 μl DMEM. Infected mice were given single dose of water or STE (250 mg/kg) daily for 14 days through intraperiteronal injection, starting form one day after infection. Infected mice were observed daily for 17 days for clinical symptoms and survival.

### Ethics

All animal methods and care described in the present study were carried out in accordance with national guide. They were approved by the Institutional Animal Care and Use Committee of National Cheng Kung University (IACUC No. 100-135).

### Statistical Analyses

Results are represented as means ± SD. All statistical analyses were computed with Graphpad Prism 5 software (GraphPad Software Inc., San Diego, California, USA). Two tailed unpaired Student’s *t* test was used to compare the mean values of two groups. The log-rank test was used to compare the survival in mice between drug-treated and water-treated group. P value less than 0.05 is considered significant.

## Electronic supplementary material

Supplementary Info
