# Supplementary material for: Antiviral activities of Schizonepeta tenuifolia Briq. against enterovirus 71 in vitro and in vivo
Source: Sci Rep. 2017 Apr 20;7:935. doi: 10.1038/s41598-017-01110-x (PMC5430552; doi:10.1038/s41598-017-01110-x)
Supplement: Supplementary file 1 — Supplementary Info [file 41598_2017_1110_MOESM1_ESM.pdf]

Antiviral activities of *Schizonepeta tenuifolia* Briq. against enterovirus 71 *in vitro* and *in vivo*

Sin-Guang Chen[1], Mei-Ling Cheng[2,3,4,5], Kuan-Hsing Chen[6], Jim-Tong Horng[7,8,9], Ching-Chuan Liu[10,12], Shih-Min Wang[10,11,12], Hiroaki Sakurai[13], Yann-Lii Leu[14,15,16] Shulhn-Der Wang[17], Hung-Yao Ho[3,5,18,*]

[1]Graduate Institute of Biomedical Science, Chang Gung University, Guishan, Taoyuan, Taiwan
[2]Department of Biomedical Sciences, College of Medicine, Chang Gung University, Guishan, Taoyuan, Taiwan
[3]Healthy Aging Research Center, Chang Gung University, Guishan, Taoyuan, Taiwan
[4]Metabolomics Core Laboratory, Chang Gung University, Guishan, Taoyuan, Taiwan
[5]Clinical Phenome Center, Chang Gung Memorial Hospital at Linkou, Guishan, Taoyuan, Taiwan
[6]Kidney Research Center, Chang Gung Memorial Hospital, Chang Gung University, School of Medicine, Taoyuan, Taiwan
[7]Department of Biochemistry, Chang Gung University, Guishan, Taoyuan, Taiwan
[8]Research Center for Emerging Viral Infections, Chang Gung University, Guishan, Taoyuan, Taiwan
[9]Molecular Infectious Disease Research Center, Chang Gung Memorial Hospital, Chang Gung University College of Medicine, Taoyuan, Taiwan
[10]Department of Pediatrics, College of Medicine, National Cheng Kung University, Tainan, Taiwan
[11]Center for Infection Control, National Cheng Kung University Hospital, Tainan, Taiwan
[12]Center of Infectious Disease and Signaling Research, National Cheng Kung University, Tainan, Taiwan
[13]Department of Cancer Cell Biology, Graduate School of Medicine and Pharmaceutical Sciences, University of Toyama, Toyama, Japan
[14]Graduate Institute of Natural Products, College of Medicine, Chang Gung University, Taoyuan, Taiwan
[15]Center for Traditional Chinese Medicine, Chang Gung Memorial Hospital at Linkou, Guishan, Taoyuan, Taiwan
[16]Chinese Herbal Medicine Research Team, Healthy Aging Research Center, Chang Gung University, Taoyuan, Taiwan
[17]School of Post-Baccalaureate Chinese Medicine, College of Chinese Medicine, China Medical University, Taichung, Taiwan

[18]Department of Medical Biotechnology and Laboratory Science, College of Medicine, Chang Gung University, Taoyuan, Taiwan
*Correspondence and requests for materials should be addressed at H.-Y. H.

Supplementary Table S1 Constituents known to be present in ST

Compound	References
**Apigenin**	32, 33
**Apigenin-7-O-β-D-glucopyranoside**	29, 31
**β-Bourbonene**	34
**Caffeic acid**	32
**Carvone**	34
**β-Caryophyllene**	30, 34
**Caryophyllene oxide**	34
**Cinnamic acid**	33
***α*-Copaene**	34
***p*-Coumaric acid**	32
***p*-Cymene-3,8-diol**	29
**Diosmetin**	27, 28
**5*α*,8*α*-Epidioxyergosta-6,22-diol-3β-ol**	29
**Hesperetin-7-O-glucoside**	33
**Hesperidin**	27-29,33
**3-Hexenyl-1-O-β-D-glucopyranoside**	29
**3-Hydroxy-4(8)-ene-*p*-menthane-3(9)-lactone**	33
**2-Hydroxy-2-isopropenyl-5-methylcyclohexanone**	34
**8-Hydroxy-*p*-menthan-3-one**	34
**Isomenthone**	34
**Limonene**	28, 30, 31, 34
**Luteolin**	27, 28, 32, 33
**Luteolin 7-O-β-D-glucopyranoside**	31
**Luteolin-7-O-β-D-glucuronopyranoside**	29
**Menthofuran**	30
**Menthone**	28, 30, 31, 34
**Mint furanone 1**	34
**β-Myrcene**	30
**1-Octen-1-ol**	34
**1-Octen-3-ol**	30, 34
**Phytol**	29
**Piperitenone**	29
**Piperitone**	34
**Pulegone**	28, 29, 30, 31, 34
**Pulegone oxide**	34
**Rosmarinic acid**	29

**Schizonepetoside A**	28, 29, 31
**Schizonepetoside B**	28, 31, 33
**Schizonepetoside C**	28, 29
**Schizonepetoside D**	28
**Schizonepetoside E**	28
**Schizonodiol**	28
**Schizonol**	28, 34
**β-Sitosterol**	29, 33
**Spatulenol**	29
**Stigmast-4-en-3-one**	29
**Tilianin**	33
**2α,3α,24α-Trihydroxyolean-12-en-28-oic acid**	29
**Ursolic acid**	29, 33

The list of compounds present in ST is compiled from references 27-34. The constituents are arranged in an alphabetical order. The corresponding references for the compounds are listed.

Supplementary Table S2 Antibodies used in this study

Product name	Cat. no.	Manufacturer or provider
Goat anti-Eps15 polyclonal antibody	sc-11716	Santa Cruz Biotechnology (Dallas, Texas, USA)
Mouse monoclonal anti-β-actin antibody	A5441	Merck Millipore (Darmstadt, Germany)
Rabbit polyclonal anti-phospho-p44/p42 MAPK (Erk 1/2) antibody	9101	Cell Signaling Technology (Danvers, MA, USA)
Rabbit polyclonal anti-p44/p42 MAPK (Erk1/2) antibody	9102	Cell Signaling Technology (Danvers, MA, USA)
Rabbit polyclonal anti-p38 MAPK antibody	9212	Cell Signaling Technology (Danvers, MA, USA)
Rabbit polyclonal anti-phospho-p38 MAPK antibody	9211	Cell Signaling Technology (Danvers, MA, USA)
Rabbit polyclonal anti-EV71-VP1 antibody	PAB7631-D01P	Abnova (Neihu District, Taipei City, Taiwan)
Rabbit polyclonal anti-eIF4G3 antibody	GTX118109	GeneTex Inc. (East District, Hsinchu City, Taiwan)
Rabbit polyclonal anti-phospho-Ser-796-Eps15 antibody		Dr. Hiroaki Sakurai
Mouse monoclonal anti-EV71-3D antibody		Dr. Shin-Ru Shih
Goat anti-rabbit IgG-HRP antibody	sc-2004	Santa Cruz Biotechnology (Dallas, Texas, USA)
Goat anti-mouse IgG-HRP antibody	sc-2005	Santa Cruz Biotechnology (Dallas, Texas, USA)
Donkey anti-goat IgG-HRP antibody	sc-2056	Santa Cruz Biotechnology (Dallas, Texas, USA)

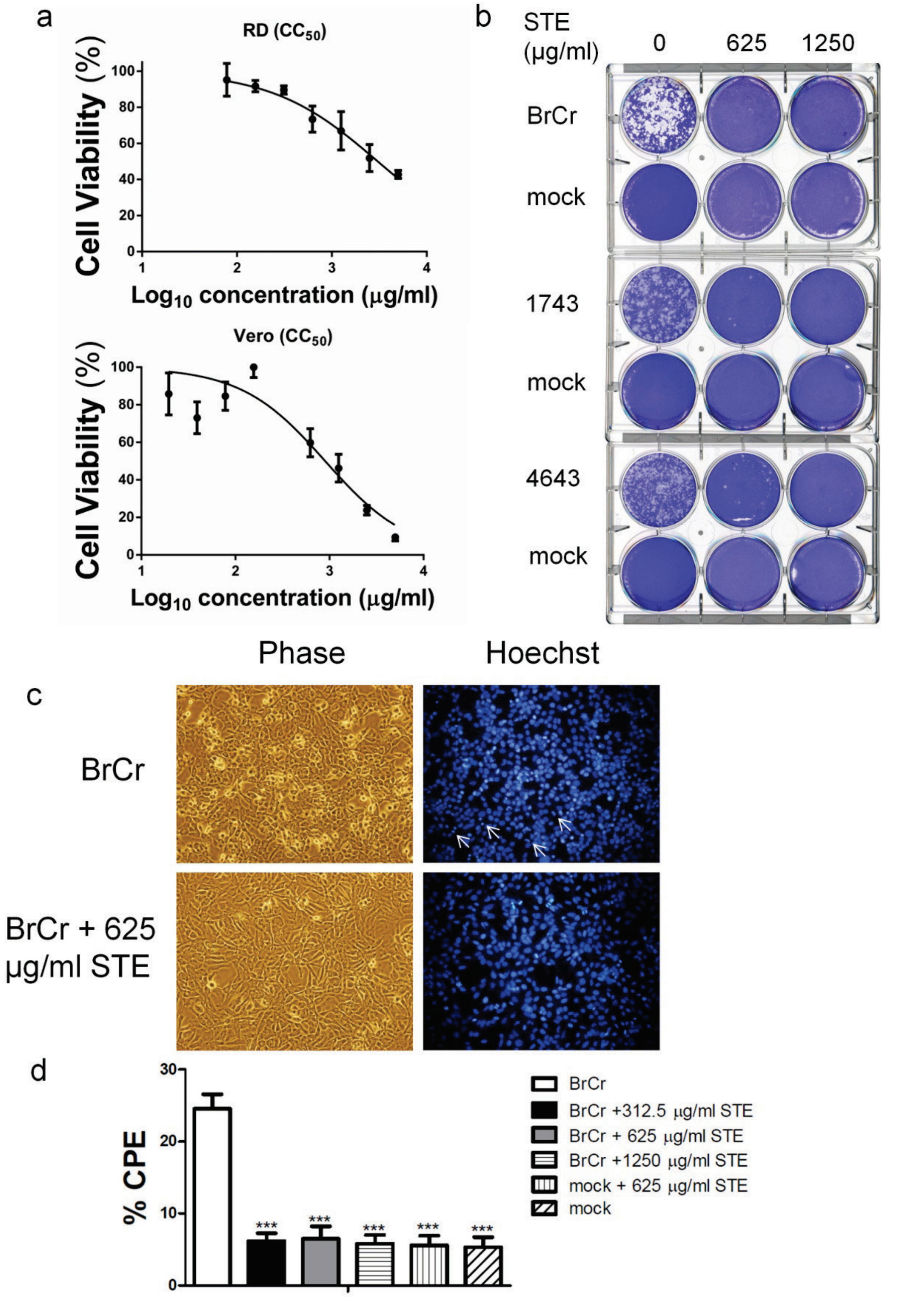

Supplementary Figure S1. STE shows a low cytotoxicity to RD and Vero cells and inhibits EV71 infection. (a) Cytotoxicity of STE to RD and Vero cells. RD and Vero cells were incubated with serially diluted STE in DMEM/2% FBS for 48 h, and the cell viability was measured with neutral red assay. Data are means±SD of three

experiments. (b) Vero cells were mock-treated or infected with 100 PFU of EV71 strains, namely BrCr, 1743 and 4643 for 1 h, and were overlaid with 0.3% agarose in DMEM/2% FBS, which was supplemented with 0, 625, or 1250 μg/ml of STE. After 96 h, the plates were fixed with 10% formalin, and stained with 1% crystal violet solution. Representative cell plates are shown here. (c) RD cells were infected with BrCr at m. o. i. of 5, and treated with indicated concentrations of STE. The cells were fixed, stained with Hoechst 33342, and observed under a fluorescence microscrope (original magnification: 100×). Virus-induced chromatin condensation and formation of crescent-shaped nuclei were observed under a fluorescence microscrope. (d) The percentage of condensed nuclei was quantified by IN Cell Analyzer 1000 (GE Heathcare Life Sciences, Chicago, USA). Data are means±SD of three experiments ***P<0.0001, vs. infected cells without treatment. The quantitative data were analyzed with the software of IN Cell Analyzer 1000.

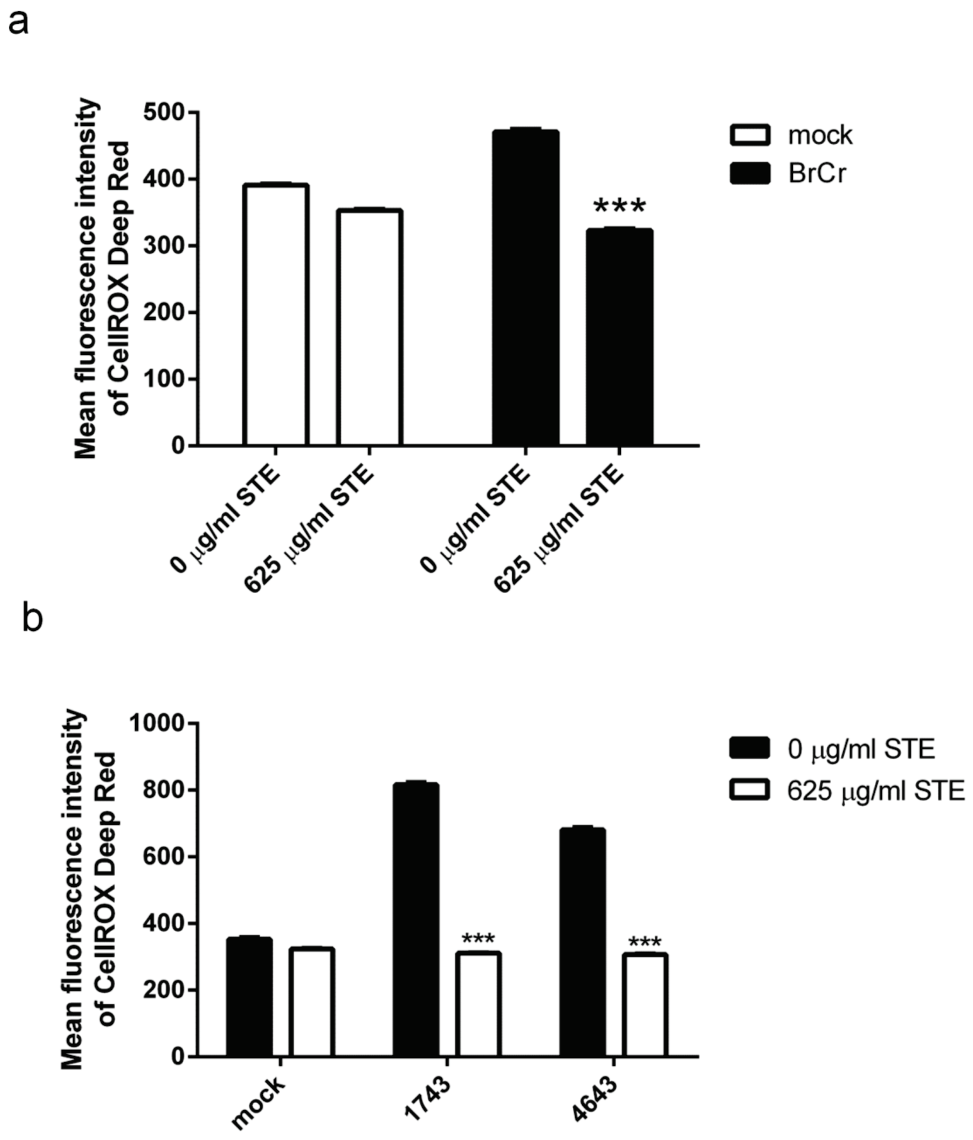

Supplementary Figure S2. STE suppresses EV71-induced ROS generation. RD cells were mock- or infected with BrCr, 1743, and 4643 at m. o. i. of 0.05 for 1 hr, and subsequently treated with indicated concentrations of STE for 24 h. Cells were loaded with CellROX Deep Red at 37 °C for 30 min, and analyzed cytometrically. Data are means±SD of six separate experiments. ***, $P<0.0001$, vs. infected cells without treatment.

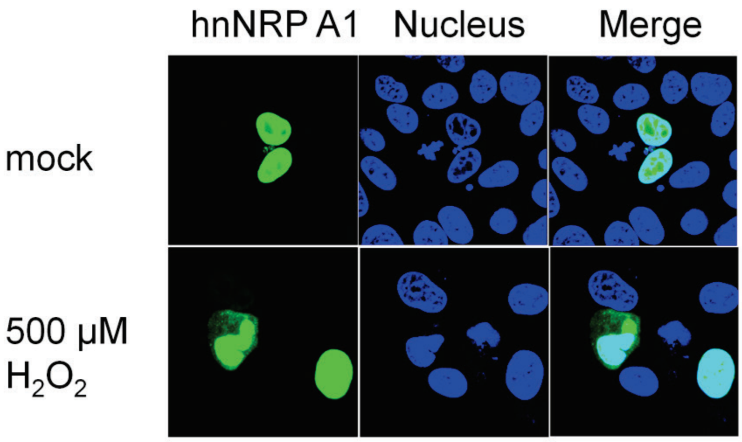

Supplementary Figure S3. Hydrogen peroxide induces the cytoplasmic accumulation of hnRNP A1. RD cells were transfected with plasmid encoding GFP-tagged hnRNP A1 for 48 h. Transfected cells were stimulated with 500 μM H_2O_2 for 7 h. The cells were fixed with 10% formalin and stained with Hoechst 33342. The image was captured with confocal microscopy. A representative result of three experiments is shown here.

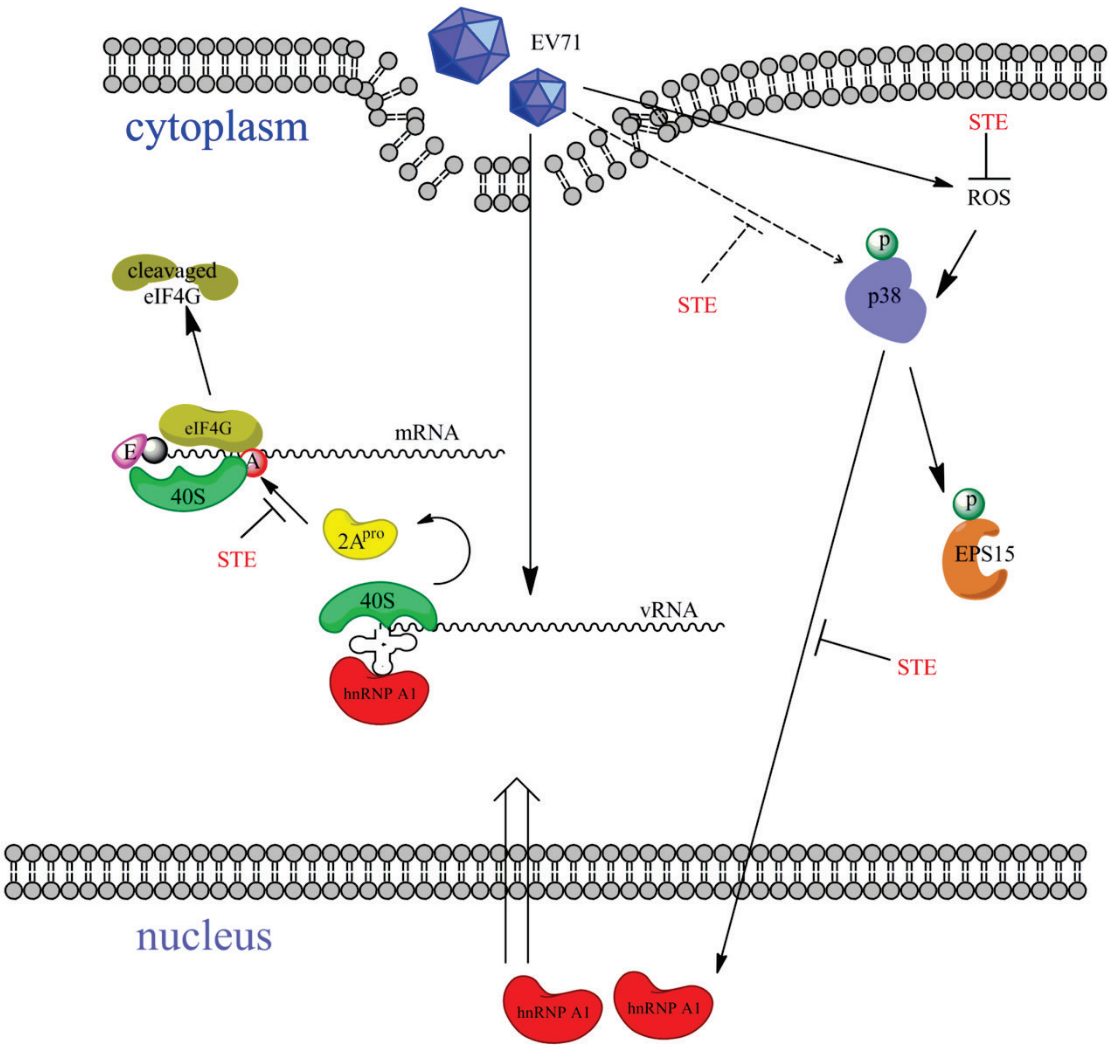

Supplementary Figure S4. A proposed model for the antiviral action of STE. In this model, EV71 binds to human scavenger receptor class B, member 2 (SCARB2), and relies on clathrin-mediated endocytosis for infectious entry. EV71-encoded protease 2A cleaves eIF4G, which serves as a scaffold of eIF4F and interacts with

cap-binding protein (eIF4E, labeled E) and helicase (eIF4A, labeled A). Concomitantly, EV71 induces generation of ROS, which activates p38 kinase. Activation of p38 kinase cascade induces translocation of hnRNP A1 from nucleus to cytoplasm to facilitate translation and replication. hnRNP A1 participates in the pathway leading to recruitment of 40S ribosome to IRES. Additionally, p38 kinase phosphorylates EPS15, which may participate in endocytosis of virus, and in membrane trafficking to promote processing of polyprotein and viral replication. The antiviral activity of STE can be attributed to its action on a number of pathways. STE interacts with virions to interfere with viral adsorption. It inhibits the protease $2A^{pro}$-mediated cleavage of eIF4G. Additionally, STE scavenges ROS, and inhibits p38 kinase activation and cytoplasmic relocation of hnRNP A1. An arrow-ending line indicates the route; a bar-ending line indicates inhibitory effect; dash line indicates an effect that cannot be ruled out in the present model.

Supplementary Methods

Cytopathic effect (CPE) assay RD cells were plated in six-well plate and incubated at 37 ℃ for 48 h. Cultured cells were washed twice with serum-free DMEM, and infected with BrCr at m. o. i. of 5 in the presence of indicated concentrations of STE. After viral absorption for 1 h, an equal volume of fresh medium containing 2% FBS and indicated concentrations of STE was added into each well. Infected cells were incubated for 6 h and fixed in 10% formalin in phosphate buffered saline (PBS). Cell nuclei were stained by 5 μg/ml of Hoechst 33342 in PBS. Virus-induced chromatin condensation and formation of crescent-shaped nuclei were observed under a fluorescence microscope, and the percentage of condensed nuclei was quantified by IN Cell Analyzer 1000 (GE Heathcare Life Sciences, Chicago, USA). The quantitative data were analyzed with the software of IN Cell Analyzer 1000.

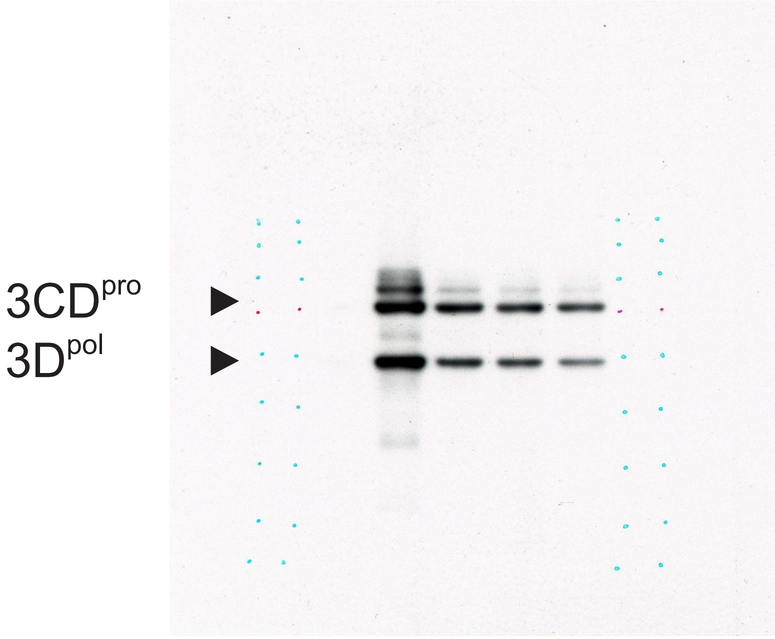

3D
(with protein markers alongside the lanes)

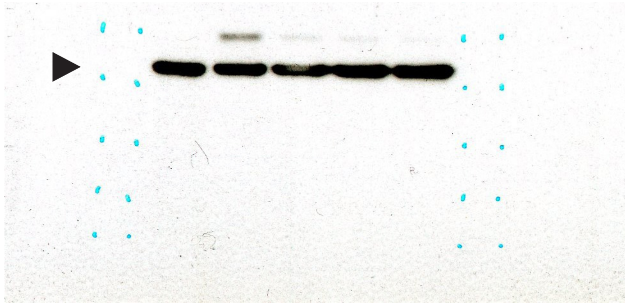

Actin (exposure time: 30 s)
(with protein markers alongside the lanes)

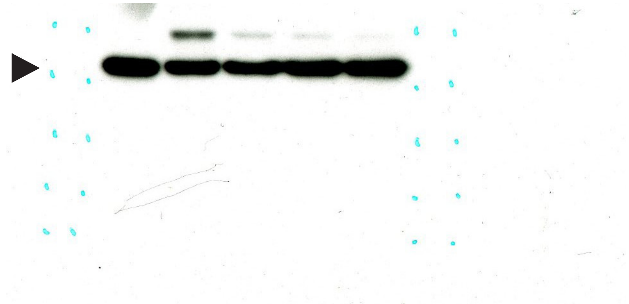

Actin (exposure time: 60 s)
(with protein markers alongside the lanes)

Raw data for the immunoblot of Figure 1c

*
*
VP1 ▶
*

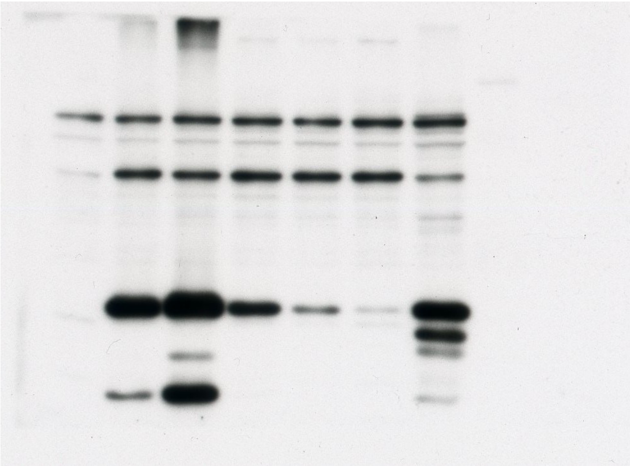

VP1 (exposure time: 60 s)

* non-specific bands

▶

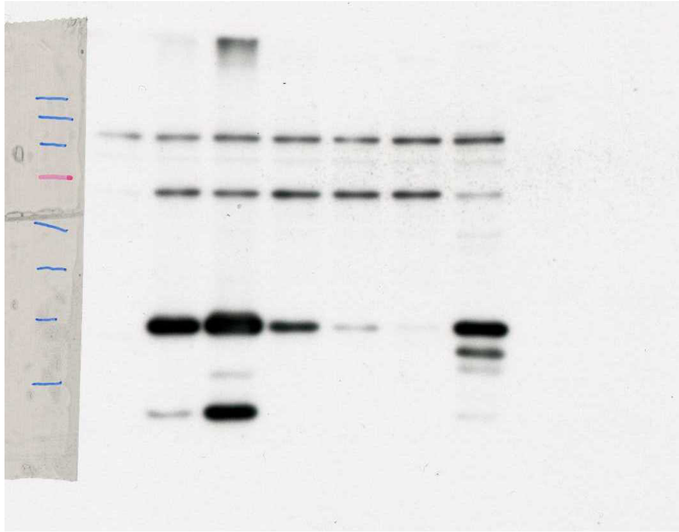

VP1 (exposure time: 30 s)

(with protein markers alongside the lanes)

Actin ▶

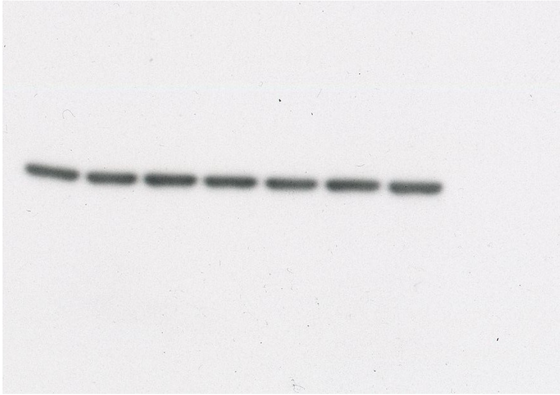

Actin

Raw data for the immunoblot of Figure 2b

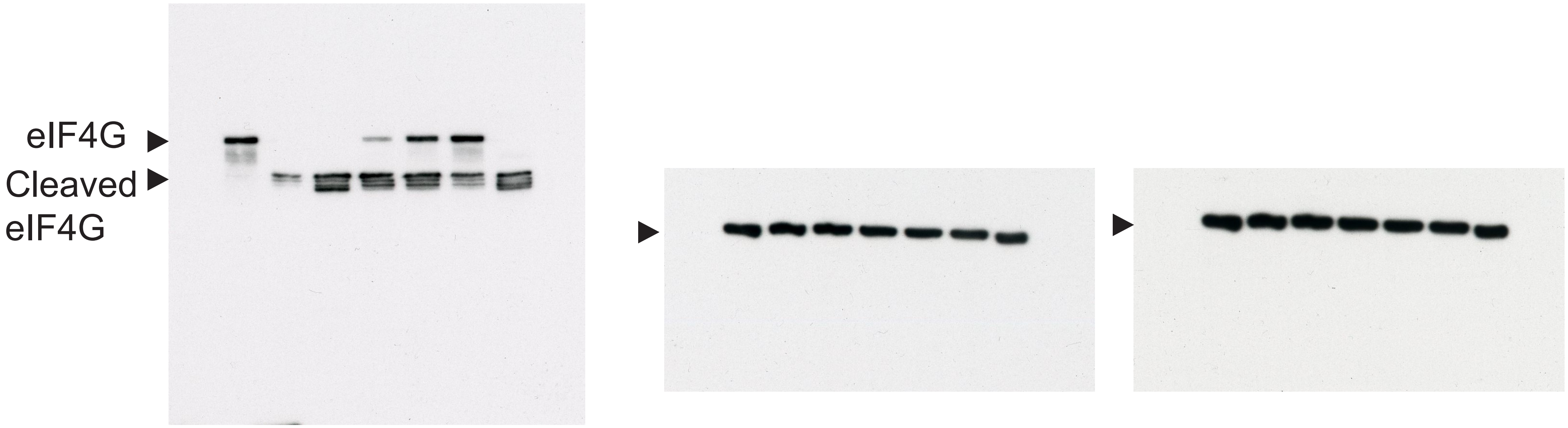

eIF4G

Actin
(exposure time: 30 s)

Actin
(exposure time: 60 s)

Raw data for the immunoblot of Figure 3c

P-p38 ▶

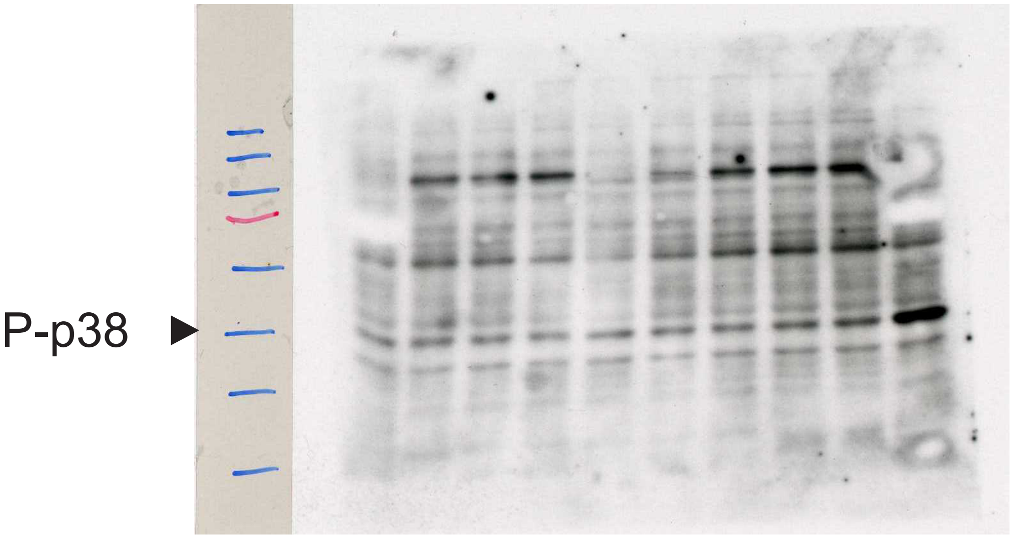

P-p38
(with protein markers alongside the lanes)

T-p38 ▶

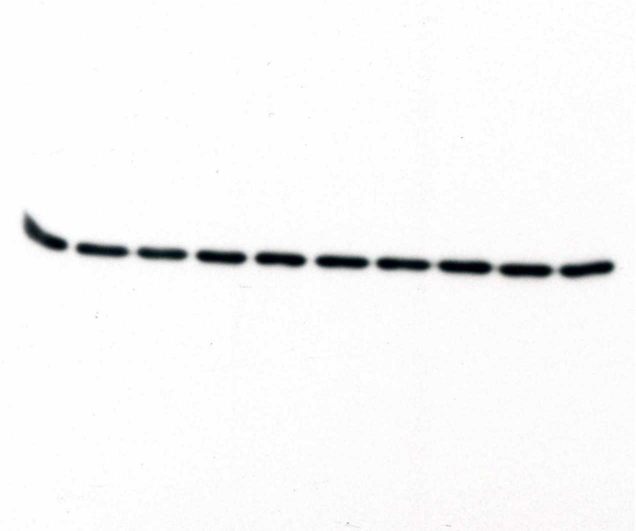

T-p38

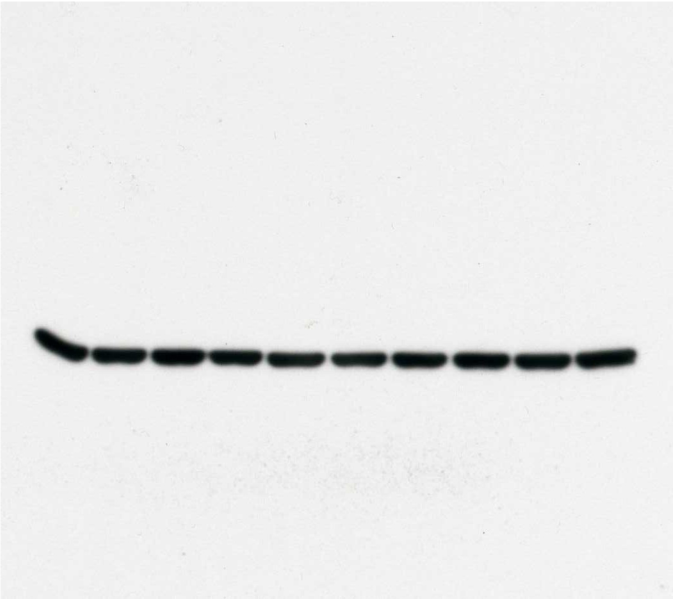

Actin (exposure time: 30 s)

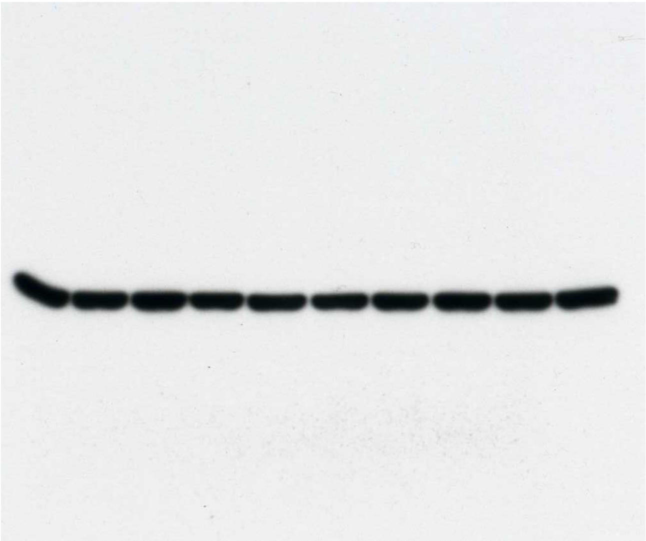

Actin (exposure time: 60 s)

Raw data for the immunoblot of the first panel of Figure 4

P-p38 ▶

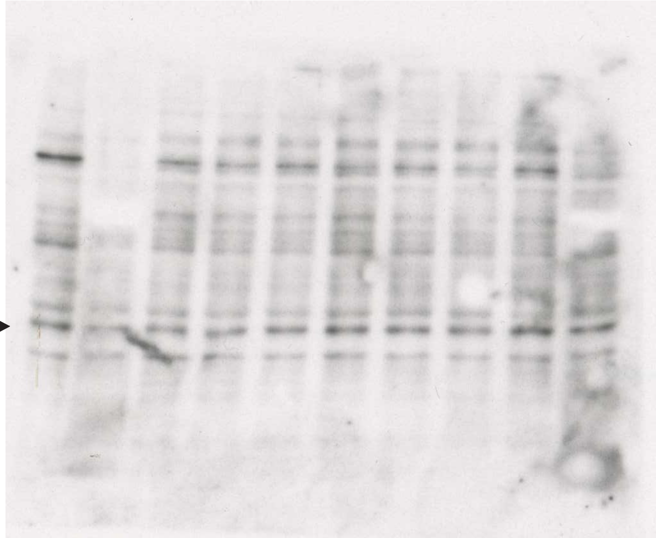

P-p38

T-p38 ▶

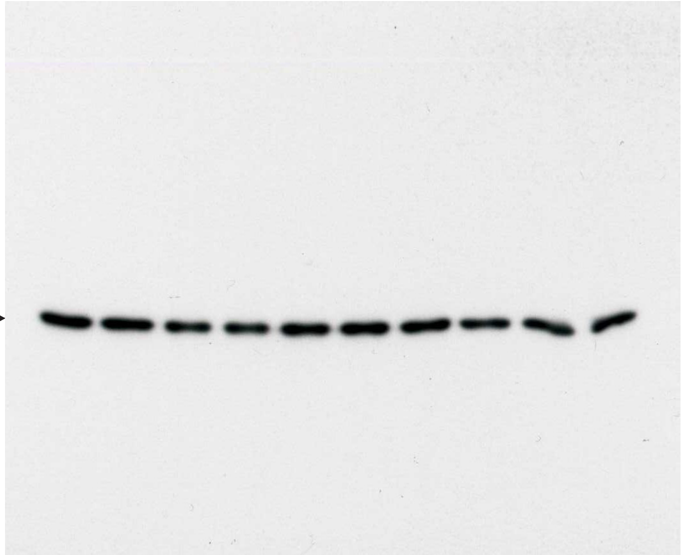

T-p38

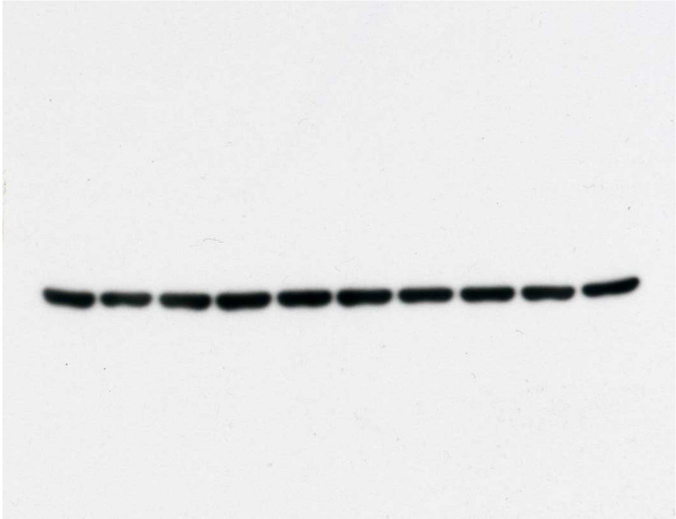

Actin (exposure time: 30 s)

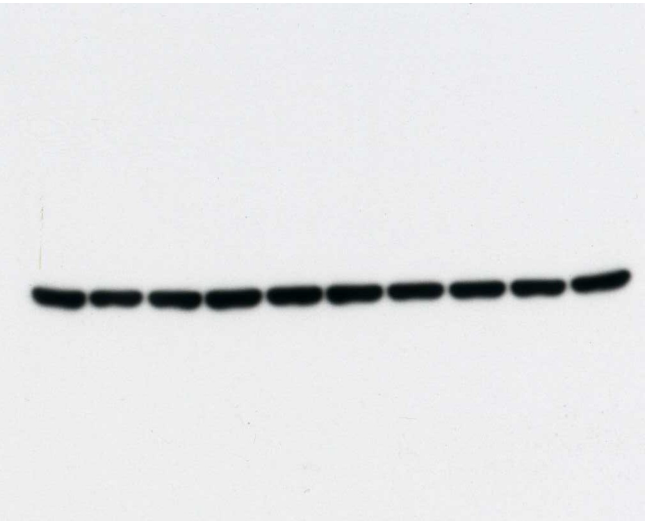

Actin (exposure time: 60 s)

Raw data for the immunoblot of the second panel of Figure 4

P-Erk ▶

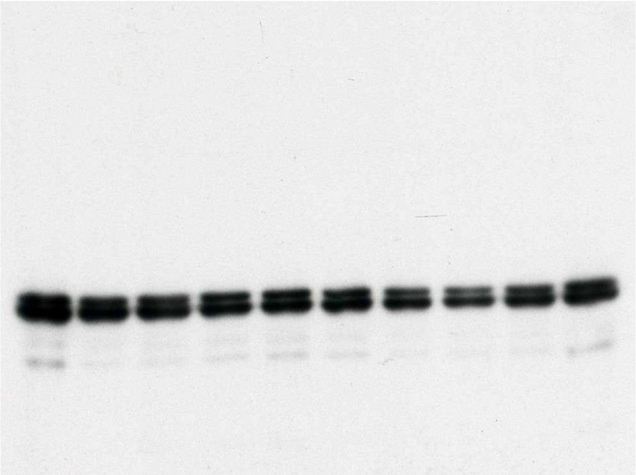

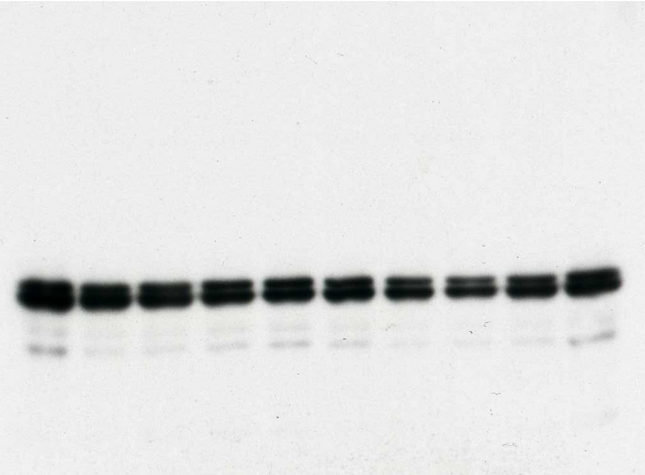

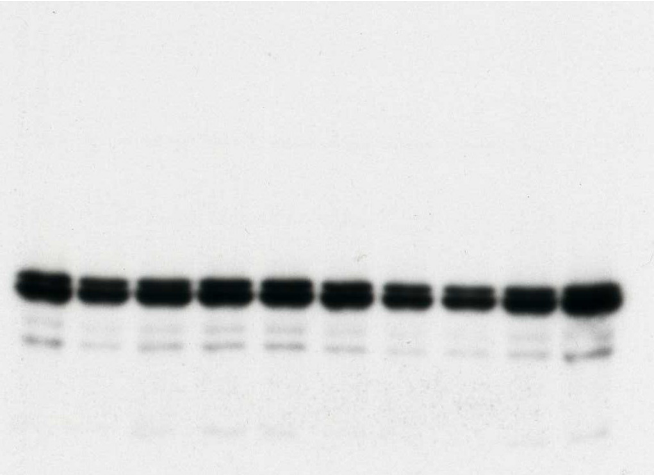

P-Erk (exposure time: 60 s) **P-Erk (exposure time: 90 s)** **P-Erk (exposure time: 120 s)**

T-Erk ▶

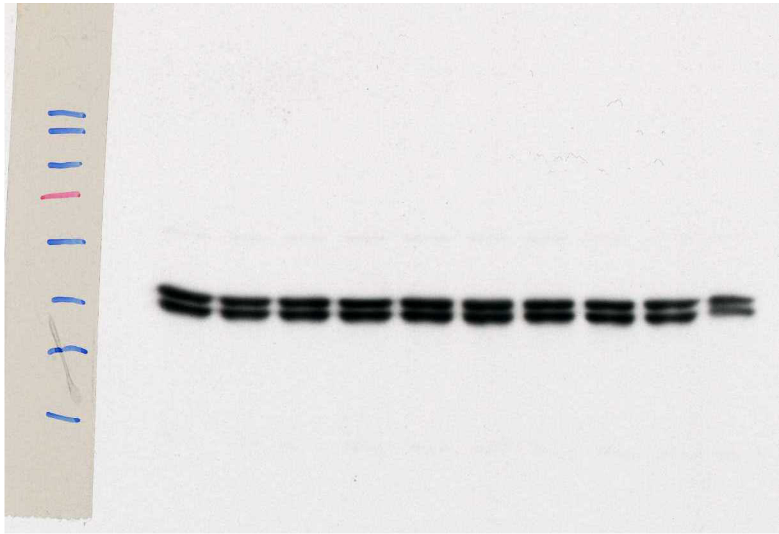

Actin ▶

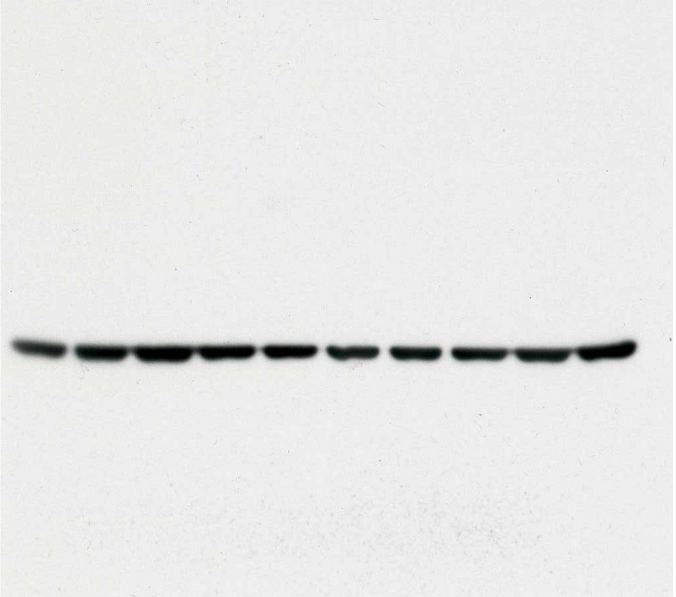

T-Erk
(with protein markers alongside the lanes)

Actin

Raw data for the immunoblot of the third panel of Figure 4

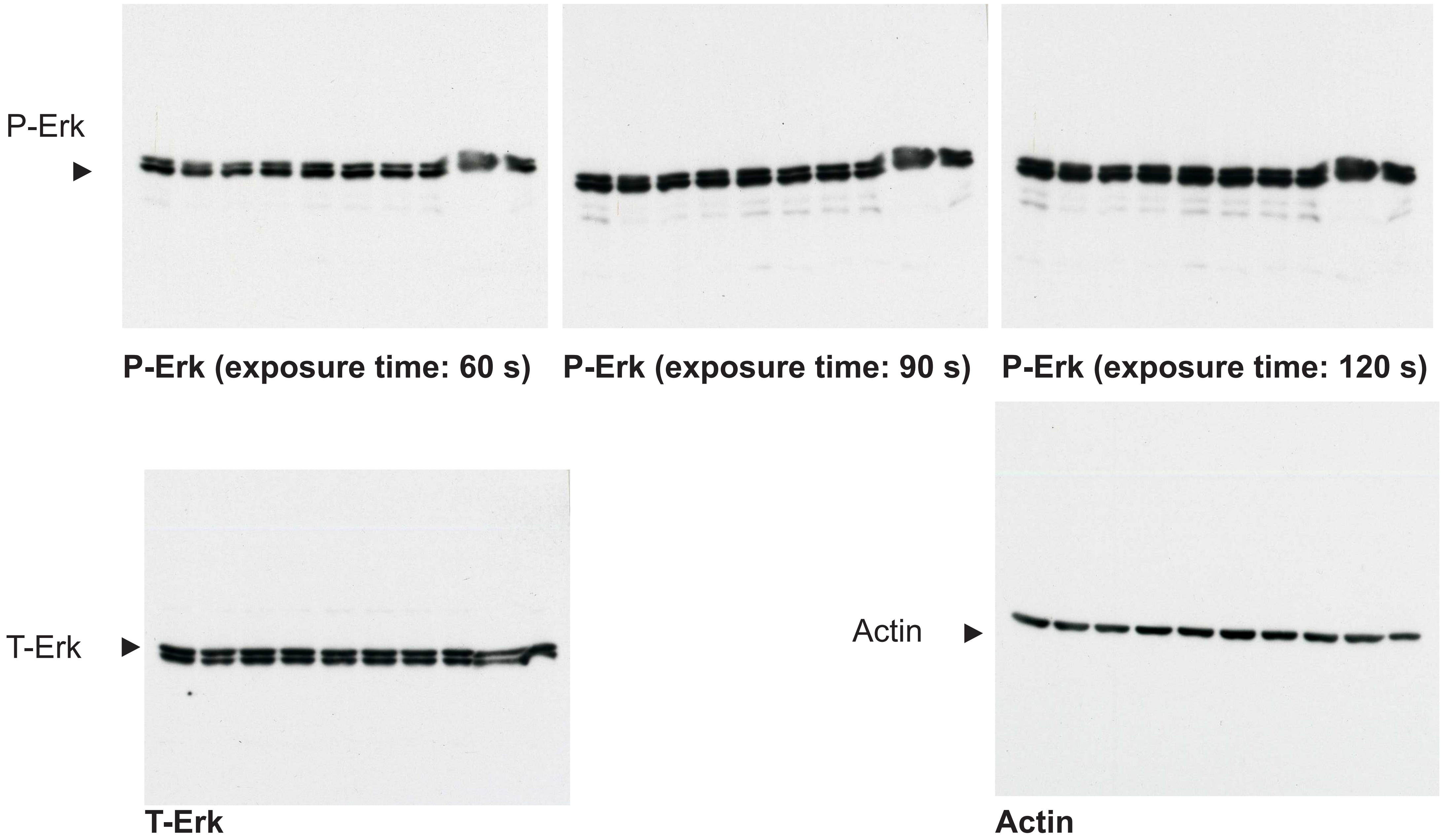

Raw data for the immunoblot of the fourth panel of Figure 4

P-p38 ▶

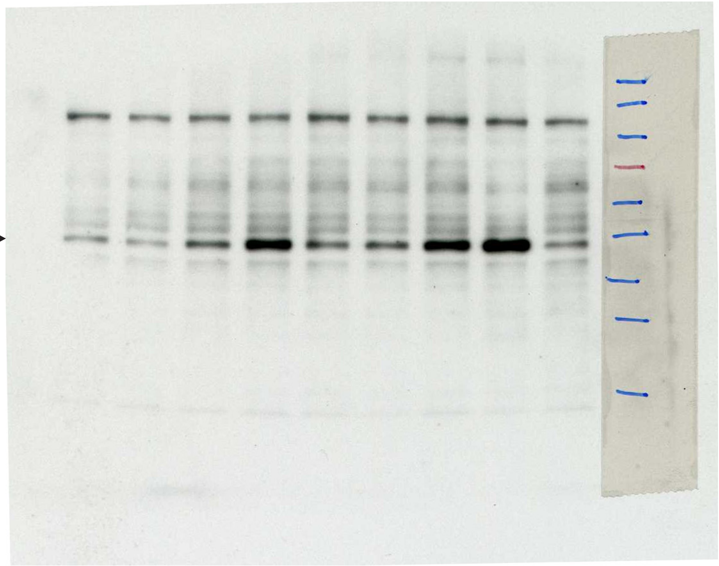

P-p38

Actin ▶

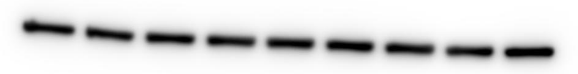

Actin

T-p38 ▶

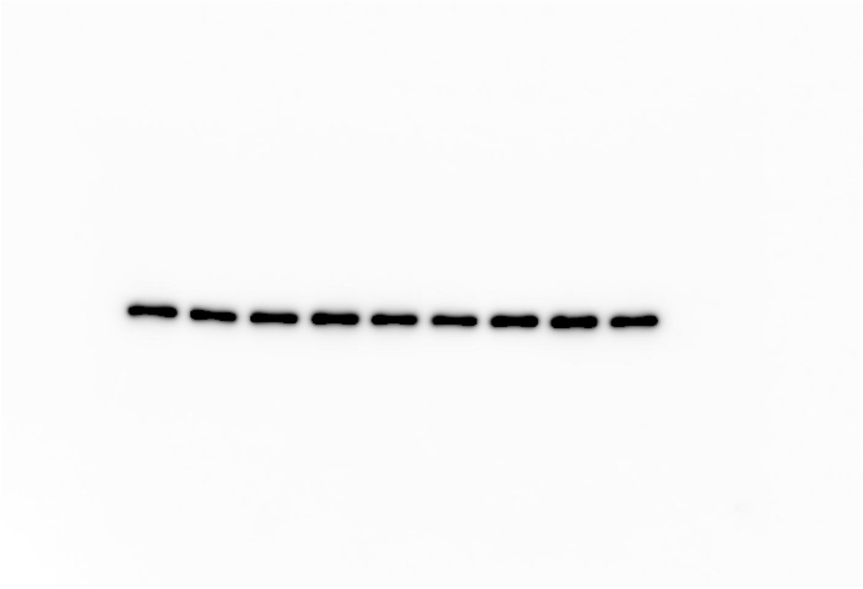

T-p38

T-p38 ▶

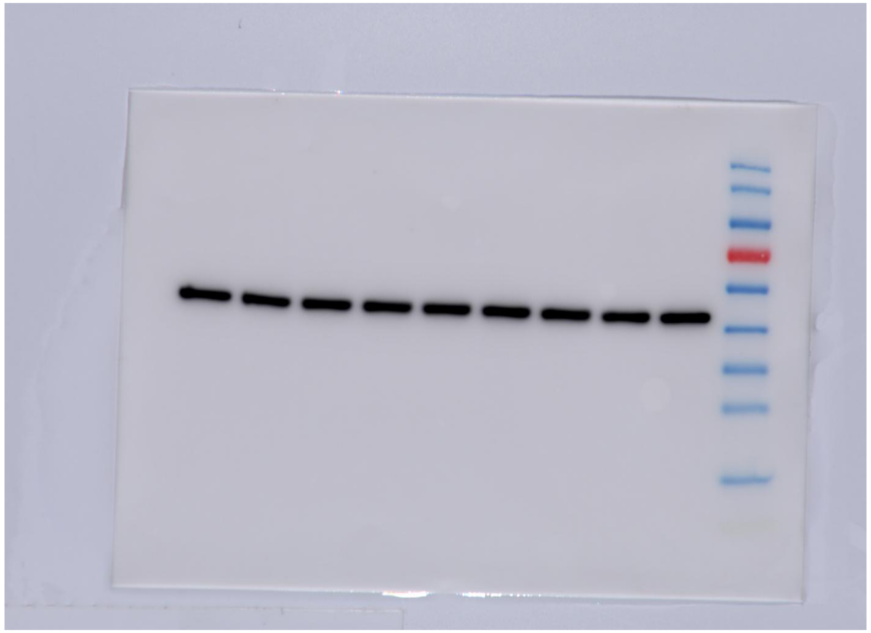

T-p38
(with protein markers alongside the lanes)

Raw data for the immunoblot of Figure 5a

VP1 ▶

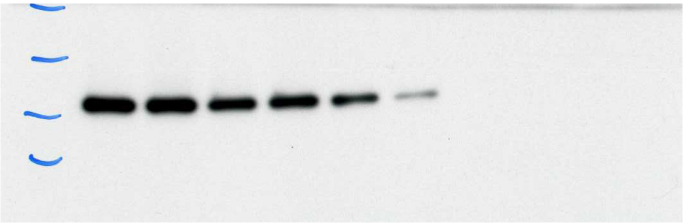

VP1
(with protein markers alongside the lanes)

eIF4G ▶
Cleaved ▶
eIF4G

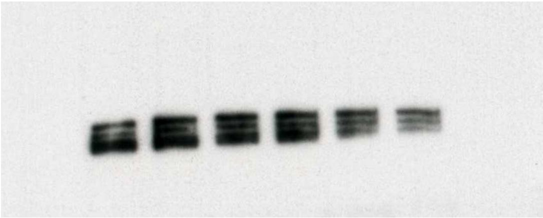

eIF4G
(exposure time: 30 s)

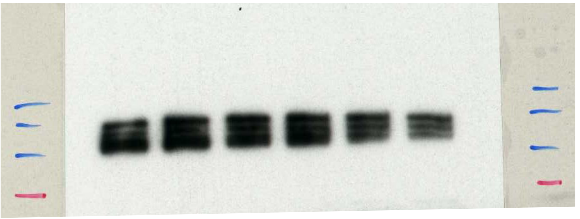

eIF4G
(exposure time: 60 s)
(with protein markers alongside the lanes)

Actin ▶

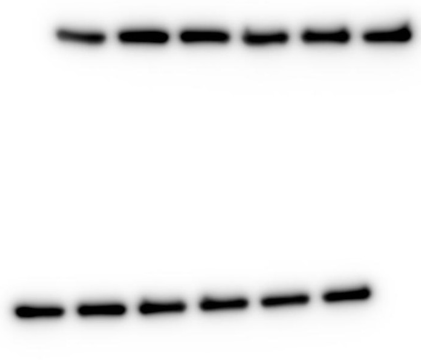

Actin

Actin ▶

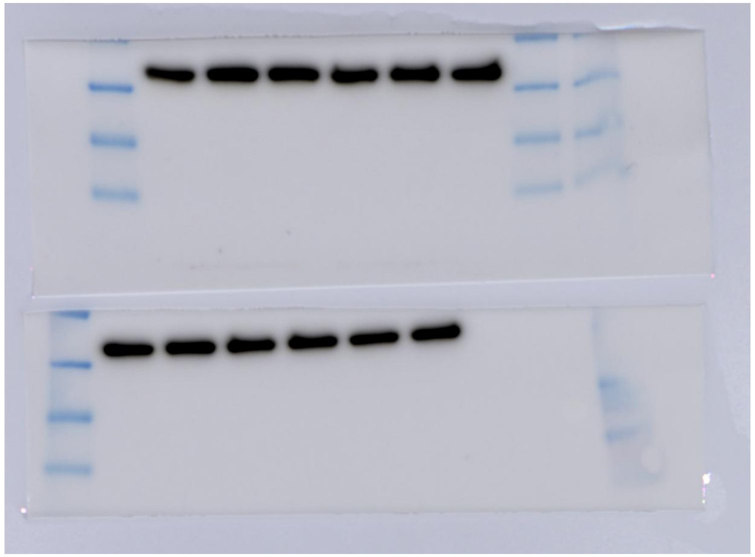

Actin
(with protein markers alongside the lanes))

Raw data for the immunoblot of Figure 5b

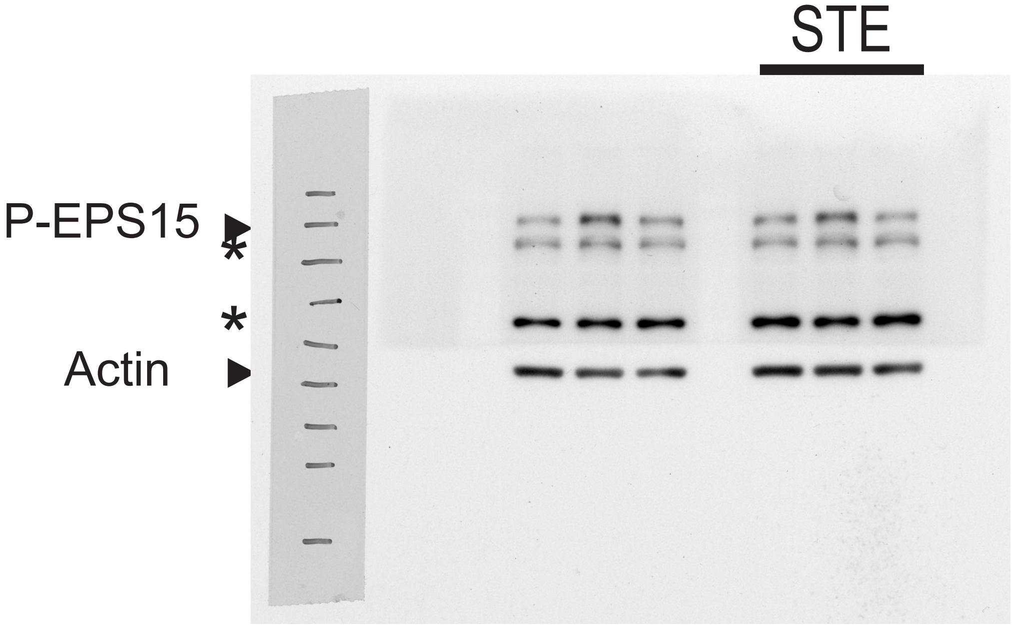

P-EPS15 and Actin

(with protein markers alongside the lanes)

It should be noted that this image represents 2 blots (i.e. blots for P-EPS15 and actin) placed side-by-side vertically.

* non-specific bands

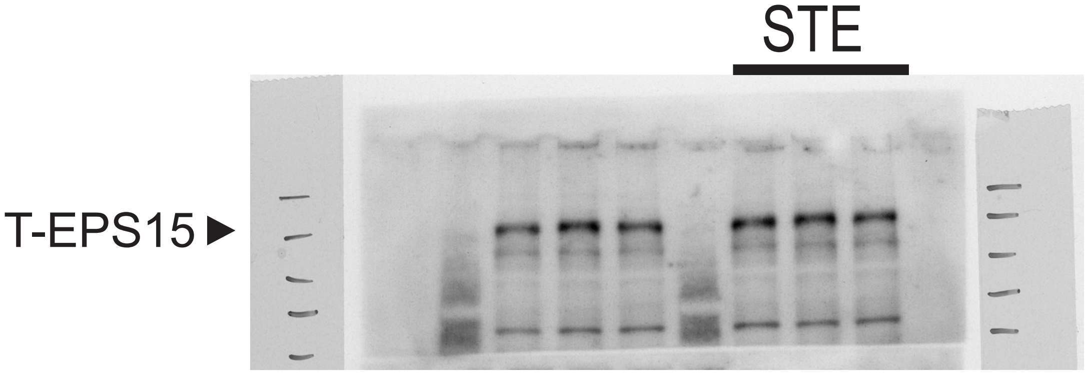

T-EPS15

(with protein markers alongside the lanes)

Raw data for the immunoblot of Figure 7a

P-EPS15 ▶
*
*

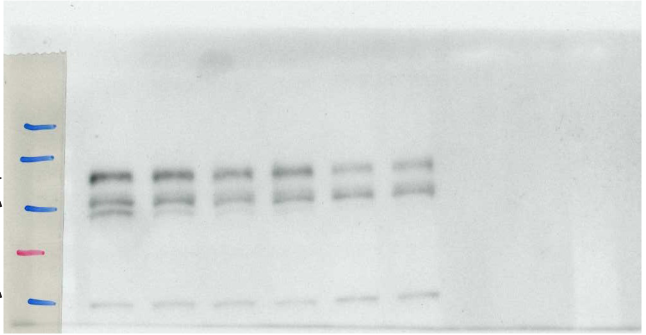

P-EPS15
(with protein markers alongside the lanes)
* non-specific bands

T-EPS15 ▶

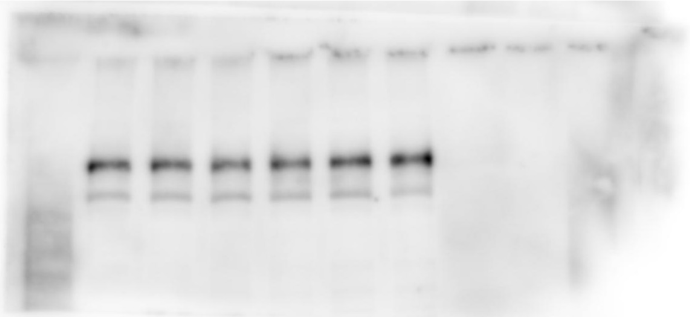

T-EPS15

Actin ▶

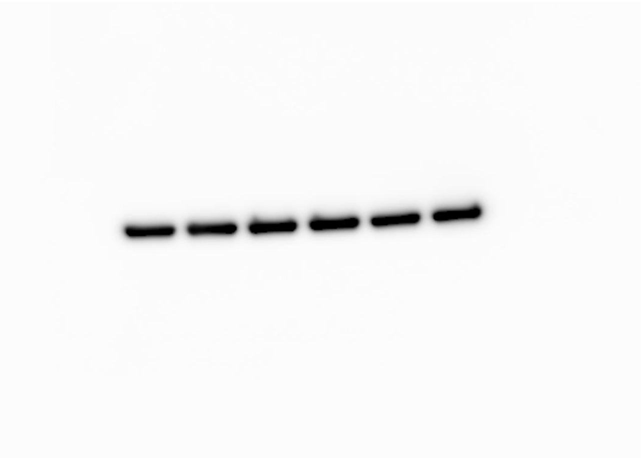

Actin (exposure time: 30 s)

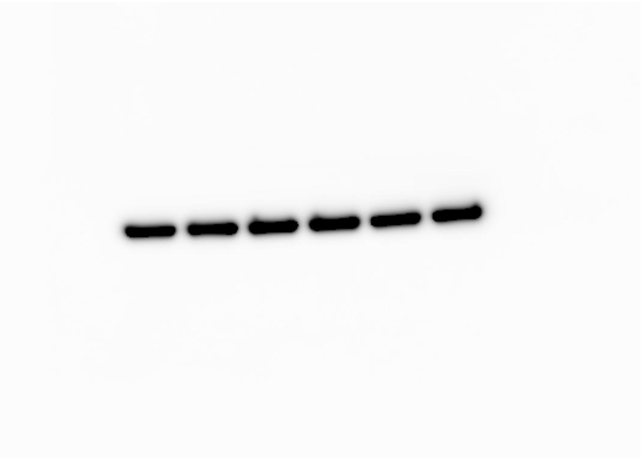

Actin (exposure time: 60 s)

Raw data for the immunoblot of Figure 7b
